# Supplementary material for: Sulfenate anions as organocatalysts for benzylic chloromethyl coupling polymerization via C=C bond formation
Source: Nat Commun. 2018 May 1;9:1754. doi: 10.1038/s41467-018-04095-x (PMC5931538; doi:10.1038/s41467-018-04095-x)
Supplement: Supplementary file 1 — Supplementary Information [file 41467_2018_4095_MOESM1_ESM.pdf]

Supplementary Information

Sulfenate Anions as Organocatalysts for Benzylic Chloromethyl Coupling Polymerization (BCCP) via C=C Bond formation

Li et al.

Supplementary Methods:

All reactions were conducted under a nitrogen atmosphere with oven-dried glassware and standard Schlenk or vacuum line techniques. All solutions were handled under nitrogen and transferred via syringe. Anhydrous solvents were purchased from Sigma-Aldrich and directly used. Unless otherwise stated, reagents were commercially available and used as purchased. Chemicals were purchased from Sigma-Aldrich, Acros, Alfa Aesar or Matrix Scientific, and solvents were purchased from Fisher Scientific and used as purchased.

In monomer synthesis, progress of reactions was monitored by thin-layer chromatography using Whatman Partisil K6F 250 μm precoated 60 Å silica gel plates and visualized by short-wave ultraviolet light as well as by treatment with iodine or ceric ammonium molybdate (CAM) stain. Flash chromatography was performed with silica gel (230–400 mesh, Silicycle).

^{1}H and $^{13}C\{^{1}H\}$ NMR spectra were obtained using a Brüker AM-500 and AM-400 Fourier-transform NMR spectrometer. Chemical shifts were reported in units of parts per million (ppm) downfield from tetramethylsilane (TMS), and all coupling constants were reported in hertz.

The infrared spectra were taken with KBr plates with a Perkin-Elmer Spectrum 100 Series spectrometer.

High resolution mass spectral analysis (HRMS) was performed on Waters-XEVOG2 Q-TOF (Waters Corporation).

The molar masses and their distribution for the polymer **P1** and **P2** were determined by GPC on an Agilent 1200 system equipped with a infinity micro vacuum degasser, (model G1379B), a Binary Pump SL (model G1312B), a High Performance Autosampler SL (model G1367D), a thermostatted column compartment SL (model G1316B), and a Diode Array Detector SL (model G1315C) using THF as eluent at a flow rate of 1 mL min^{-1} on a Agilent PL1113-6300 resipore (300 × 75 mm) column at 40 ºC. The system was calibrated with Agilent polystyrene medium Easivials (162–500000 g/mol) calibration kit.

The molar masses and their distribution for the polymer **P3-1** and **P4-1** were determined by GPC on a Waters system equipped with a set of three Ultrastyragel columns (HT2, HT3, and HT4; 30 cm × 7.8 mm; 10 μm particles; exclusion limits: 100-10000, 500-30000, and 5000-600000 g/mol, respectively), Waters 515 HPLC pump, Waters 717 plus autosampler and an online Waters 2414 refractive index detector maintained at 35°C. THF was used as the mobile phase (1 mL/min), and polystyrene samples as the standards in the calibration of the molar masses.

Preparation of monomer M1:

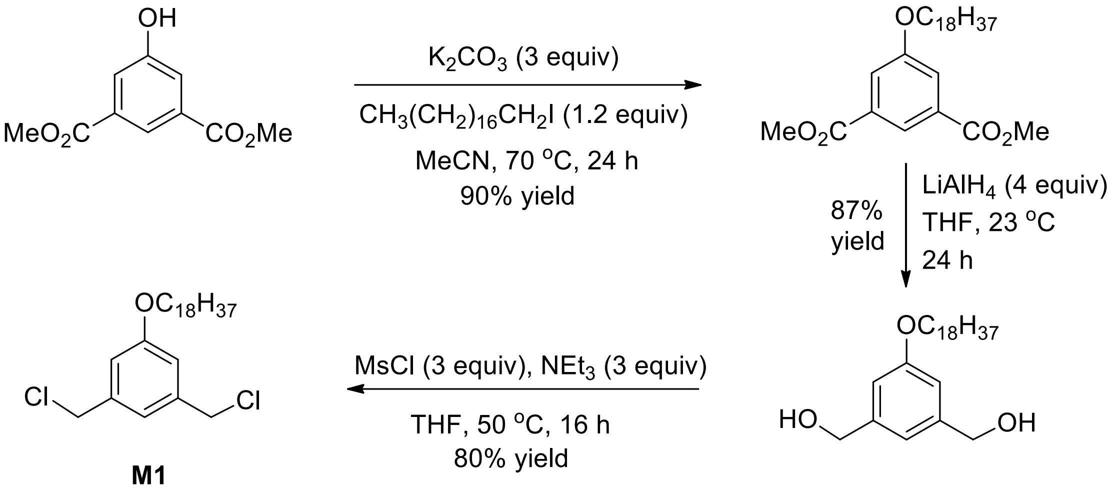

M1

Dimethyl 5-(octadecyloxy)benzene-1,3-dioate

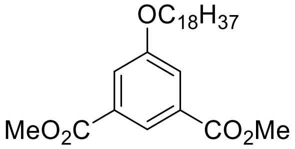

An oven-dried 250 mL Schlenk tube equipped with a stir bar was charged with a mixture of dimethyl 5-hydroxybenzene-1,3-dioate (6.3 g, 30.0 mmol), 1-iodooctadecane (13.7 g, 36.0 mmol) and K_2CO_3 (12.4 g, 90.0 mmol). MeCN (50 mL) was added via syringe through the rubber septum. The reaction mixture was then heated to 70 °C for 24 h. After cooling to room temperature, the mixture was diluted with water (100 mL) and was extracted with EtOAc (50 mL × 3). The combined organic layers were washed with saturated brine (200 mL), then dried over anhydrous Na_2SO_4 and concentrated, dried under vacuum to afford dimethyl 5-(octadecyloxy)benzene-1,3-dioate in 12.47 g, 90% yield as a white solid.

m.p. 63–65 °C. R_f = 0.59 (hexanes : ethyl acetate = 9 : 1). ^{1}H NMR (500 MHz, $CDCl_3$): δ 8.26 (t, *J* = 1.5, 1H), 7.74 (d, *J* = 1.5, 2H), 4.03 (t, *J* = 6.5, 2H), 3.94 (s, 6H), 1.83–1.77 (m, 2H), 1.48–1.44 (m, 2H), 1.26 (m, 28H), 0.89 (t, J = 6.5, 3H) ppm; ^{13}C{^{1}H} NMR (125 MHz, $CDCl_3$): δ 166.4, 159.5, 131.9, 122.9, 120.0, 68.8, 52.5, 32.1, 29.91, 29.89, 29.87, 29.80, 29.77, 29.57, 29.56, 29.3, 26.2, 22.9, 14.3 ppm. IR (thin film): 3436, 2914, 2840, 1730, 1471, 1346, 1253, 754 cm^{-1}; HRMS calc'd for $C_{28}H_{46}O_5^+$ 462.3345, observed 462.3322 [M-C_2H_4]$^+$.

1,3-Bis(hydroxymethyl)-5-(octadecyloxy)benzene

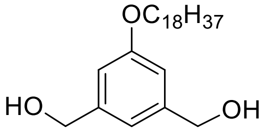

An oven-dried 100 mL Schlenk tube equipped with a stir bar was charged with dimethyl 5-(octadecyloxy)benzene-1,3-dioate (2.4 g, 5.2 mmol). The Schlenk tube was sealed with a rubber septum, connected to a Schlenk line, and evacuated and refilled with nitrogen (repeated three times). Anhydrous THF (15 mL) was added under nitrogen via syringe through the rubber septum and the solution was stirred at 0 ^oC for 30 min. Next, 10.4 mL of $LiAlH_4$ (20.8 mmol, 2.0 M solution in THF) was added dropwise into the Schlenk tube via syringe. The mixture was warmed to room temperature and stirred for 24 h under nitrogen. After cooling to 0 ^oC for 30 mins, an aqueous solution of HCl (22 mL, 1 M) was added slowly. The mixture was diluted with ethyl acetate (20 mL), the layers were separated, the organic layer was washed with saturated brine (20 mL) and the mixture was concentrated, dried under vacuum to afford 1,3-bis(hydroxymethyl)-5-(octadecyloxy)benzene in 1.84 g, 87% yield as a white solid.

m.p. 81–83 ^oC. R_f = 0.27 (hexanes : ethyl acetate = 1 : 1). ^1H NMR (500 MHz, THF-d_8): δ 6.84–6.83 (m, 1H), 6.76–6.75 (m, 2H), 4.50 (d, *J* = 4.5, 4H), 4.06–4.04 (m, 2H), 3.94 (t, *J* = 6.5, 2H), 1.77–1.73 (m, 2H), 1.50–1.44 (m, 2H), 1.29 (m, 28H), 0.89 (t, J = 6.5, 3H) ppm; $^{13}C\{^1H\}$ NMR (125 MHz, THF-d_8): δ 160.6, 145.2, 117.3, 111.7, 68.5, 65.1, 33.0, 30.8, 30.80, 30.79, 30.77, 30.6, 30.54, 30.48, 27.6, 25.9, 23.7, 14.6 ppm. IR (thin film): 2915, 2850, 913, 747 cm^{-1}; HRMS calc'd for $C_{26}H_{46}O_3Na^+$ 429.3345, observed 429.3346 $[M+Na]^+$.

1,3-Bis(chloromethyl)-5-(octadecyloxy)benzene (M1)

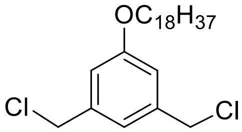

An oven-dried 250 mL round bottom flask equipped with a stir bar was charged with 1,3-bis(hydroxymethyl)-5-(octadecyloxy)benzene (1.4 g, 3.45 mmol). Chloroform (50 mL) was added via syringe and the solution was cooled to 0 ^oC. Methane sulfonyl chloride (0.8 mL, 10.35 mmol) and triethylamine (1.5 mL, 10.35 mmol) was added dropwise into the round bottom flask via syringe at 0 ^oC. The, reaction vessel was sealed with a septum, put in an oil bath, stirred at 50 ^oC for 16 h and cooled to room temperature. After quenching the reaction mixture with water (20 mL) the layers were separated. The reaction mixture was then extracted with chloroform (10 mL × 3). The combined organic phases was, dried over anhydrous Na_2SO_4 and concentrated. The residue was purified by column chromatography (silica gel, hexanes to hexanes : ethyl acetate = 20 : 1) to afford 1.22 g of the momomer **1** (80% yield) as a white solid.

m.p. 59–60 °C. R_f = 0.75 (hexanes : ethyl acetate = 9 : 1). ^{1}H NMR (500 MHz, $CDCl_3$): δ 6.97 (s, 1H), 6.87 (s, 2H) , 4.53 (s, 4H), 3.96 (t, *J* = 6.0, 2H), 1.77 (m, 2H), 1.46 (m, 2H), 1.27 (m, 28H), 0.88 (m, 3H) ppm; $^{13}C\{^1H\}$ NMR (125 MHz, $CDCl_3$): 159.9, 139.5, 120.8, 114.9, 68.4, 46.1, 32.2, 29.9, 29.89, 29.82, 29.79, 29.6, 29.4, 26.2, 22.9, 14.3 ppm. IR (thin film): 2926, 2854, 2359, 2340, 969, 738 cm^{-1}; HRMS calc'd for $C_{26}H_{44}OCl_2^+$ 442.2769, observed 442.2782 [M+H]$^+$.

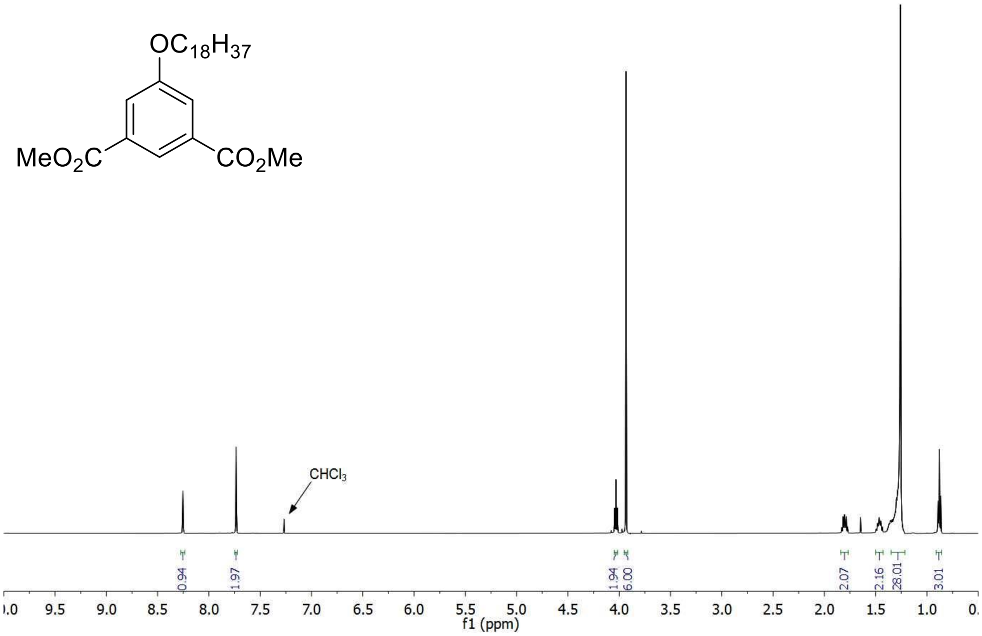

Supplementary Figure 1. Dimethyl 5-(octadecyloxy)benzene-1,3-dioate

(^{1}H NMR spectra, $CDCl_3$, 500 MHz)

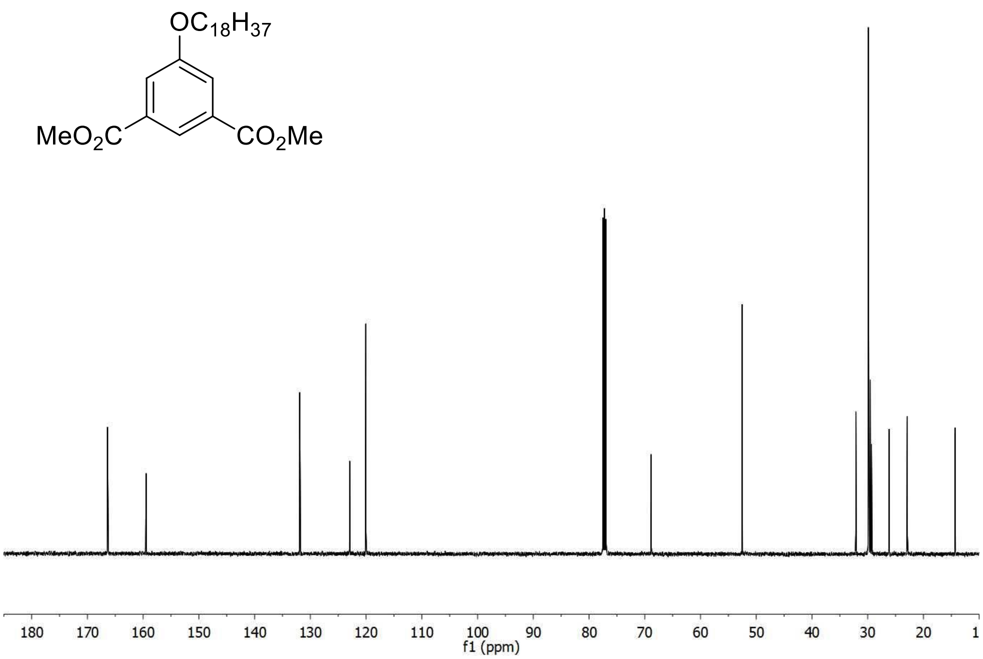

Supplementary Figure 2. Dimethyl 5-(octadecyloxy)benzene-1,3-dioate

($^{13}C\{^{1}H\}$ NMR spectra, $CDCl_3$, 125 MHz)

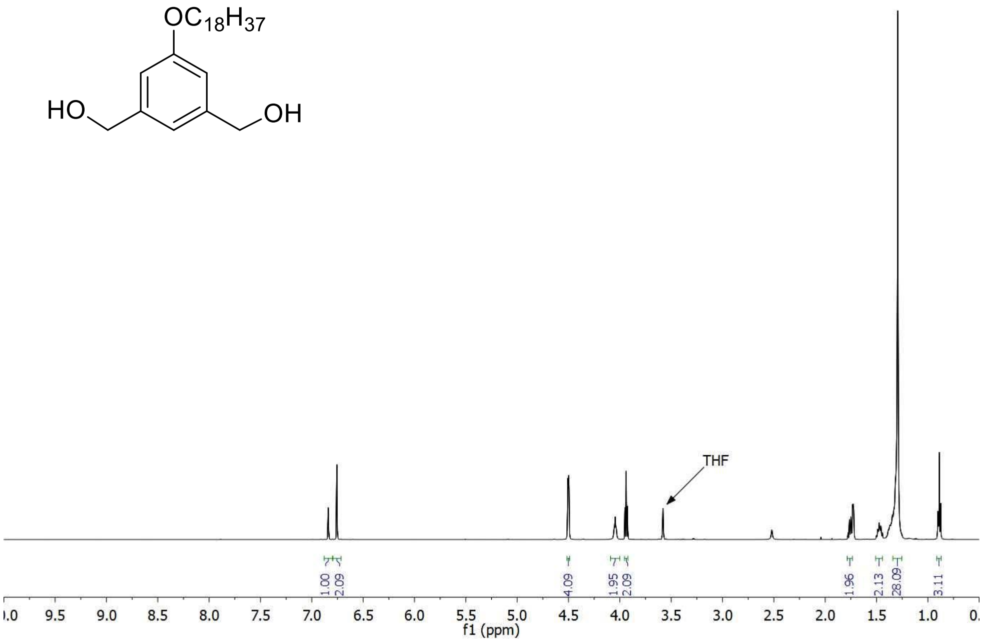

Supplementary Figure 3. 1,3-Bis(hydroxymethyl)-5-(octadecyloxy)benzene

(^{1}H NMR spectra, THF-d_8, 500 MHz)

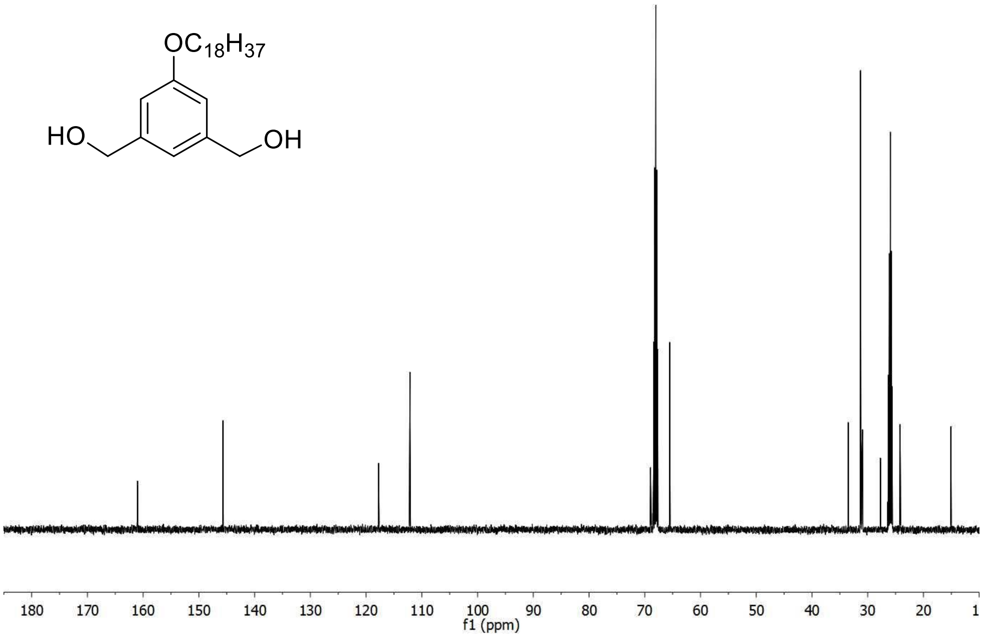

Supplementary Figure 4. 1,3-Bis(hydroxymethyl)-5-(octadecyloxy)benzene

($^{13}C\{^{1}H\}$ NMR spectra, THF-d_8, 125 MHz)

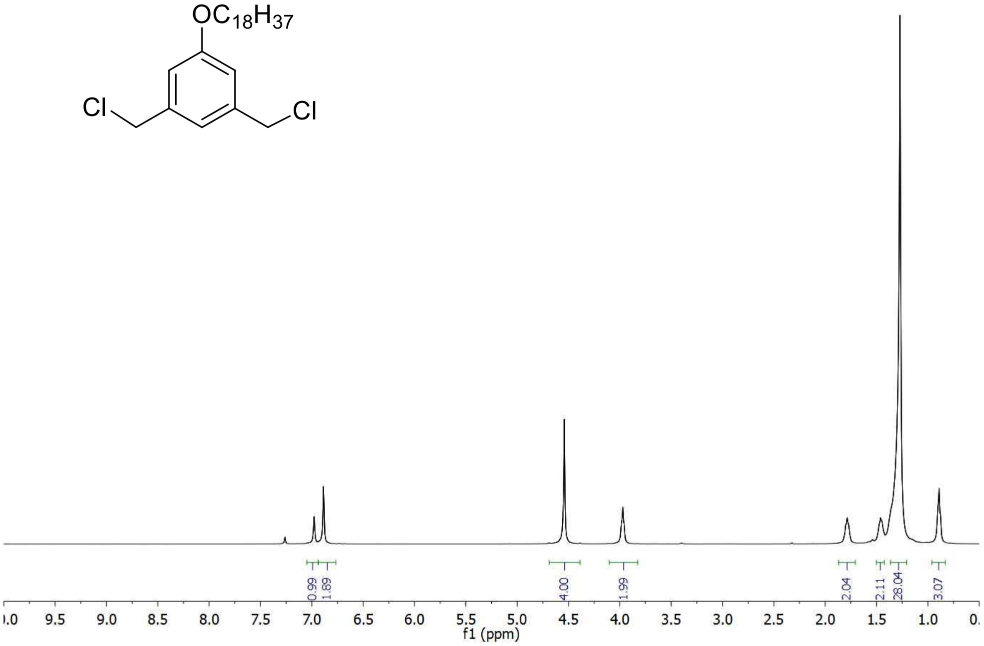

Supplementary Figure 5. 1,3-Bis(chloromethyl)-5-(octadecyloxy)benzene (M 1)

(^{1}H NMR spectra, $CDCl_3$, 500 MHz)

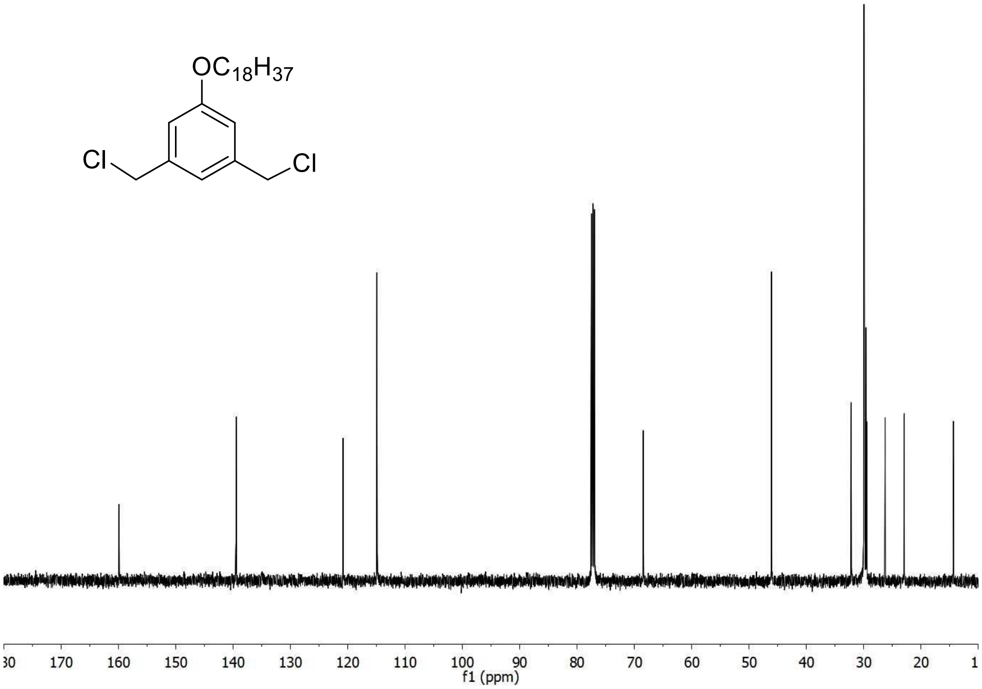

Supplementary Figure 6. 1,3-Bis(chloromethyl)-5-(octadecyloxy)benzene (M 1)

($^{13}C\{^{1}H\}$ NMR spectra, $CDCl_3$, 125 MHz)

Preparation of monomer M2:

4,4'-(Perfluoropropane-2,2-diyl)bis((chloromethyl)benzene) (M2)

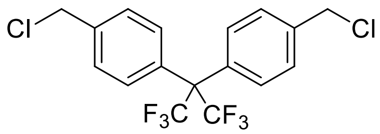

An oven-dried 100 mL Schlenk tube equipped with a stir bar was charged with dimethyl 4,4'-(perfluoropropane-2,2-diyl) dibenzoic acid (2.0 g, 5.0 mmol). The Schlenk tube was sealed with a rubber septum, connected to a Schlenk line, and evacuated and refilled with nitrogen (repeated three times). Anhydrous THF (15 mL) was added under nitrogen via syringe through the rubber septum and the solution was stirred at 0 °C for 30 min. Next, 10.0 mL of $LiAlH_4$ (20.0 mmol, 2.0 M solution in THF) was added dropwise into the Schlenk tube via syringe. The mixture was warmed to room temperature and stirred for 24 h under nitrogen. After cooling to 0 °C, an aqueous solution of HCl (22 mL, 1 M) was added slowly. The mixture was diluted with ethyl acetate (20 mL), the layers were separated, the organic layer was washed with saturated brine (20 mL), dried over anhydrous Na_2SO_4 and mixture was concentrated in a 250 mL round bottom flask. The mixture was next dried under vacuum for 12 h and used as obtained in the next step.

A stir bar was added to the round bottom flask. Chloroform (50 mL) was added via syringe and the solution was cooled to 0 °C. Methane sulfonyl chloride (1.2 mL, 15.1 mmol) and triethylamine (2.2 mL, 15.1 mmol) was added dropwise into the round bottom flask via syringe at 0 °C. The resulting mixture was sealed, then put in an oil bath and stirred at 50 °C for 16 h and then cooled to room temperature. After quenching the reaction with water (20 mL), the layers were separated. The reaction mixture was extracted with chloroform (10 mL × 3). The combined organic phase was dried over anhydrous Na_2SO_4 and concentrated. The residue was purified by column chromatography (silica gel, hexanes) to afford 1.30 g of the momomer **2** (65% yield over 2 steps).

m.p. 83-85 °C. R_f = 0.18 (hexanes). ^{1}H NMR (500 MHz, $CDCl_3$): δ 7.42–7.37 (m, 8H), 4.59 (s, 4H) ppm; $^{13}C\{^1H\}$ NMR (125 MHz, $CDCl_3$): 138.7, 133.6, 130.8, 128.6, 124.2 (d, $J_{C\text{-}F}$ = 284 Hz), 64.5, 45.4 ppm. IR (thin film): 2354, 1770, 1517, 1447, 1250, 1174, 869, 772 cm^{-1}; HRMS calc'd for $C_{17}H_{13}F_6Cl_2^+$ 400.0220, observed 400.0211 $[M+H]^+$.

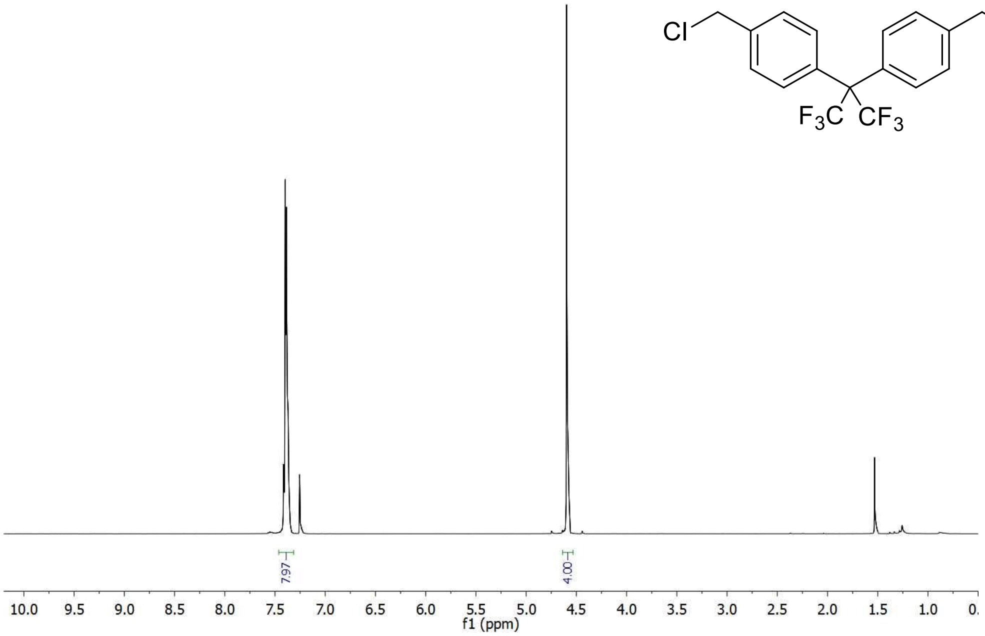

Supplementary Figure 7. 4,4'-(perfluoropropane-2,2-diyl)bis((chloromethyl)benzene) (M 2)

(^{1}H NMR spectra, $CDCl_3$, 500 MHz)

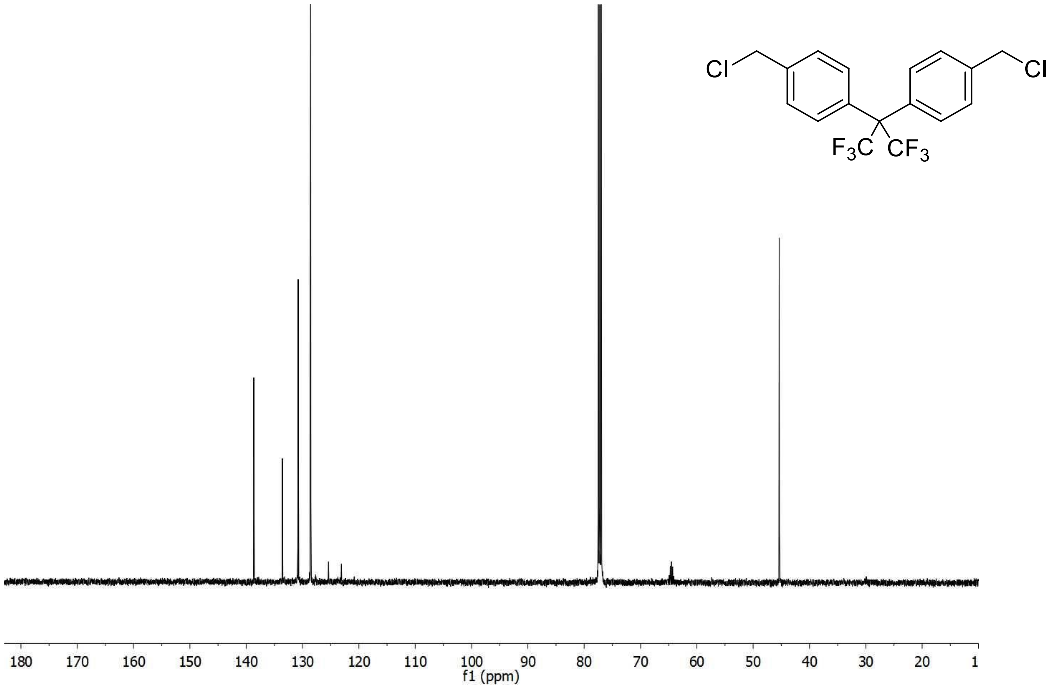

Supplementary Figure 8. 4,4'-(perfluoropropane-2,2-diyl)bis((chloromethyl)benzene) (M2)

(^{13}C{^{1}H} NMR spectra, $CDCl_3$, 125 MHz)

Preparation of monomer M3-1:

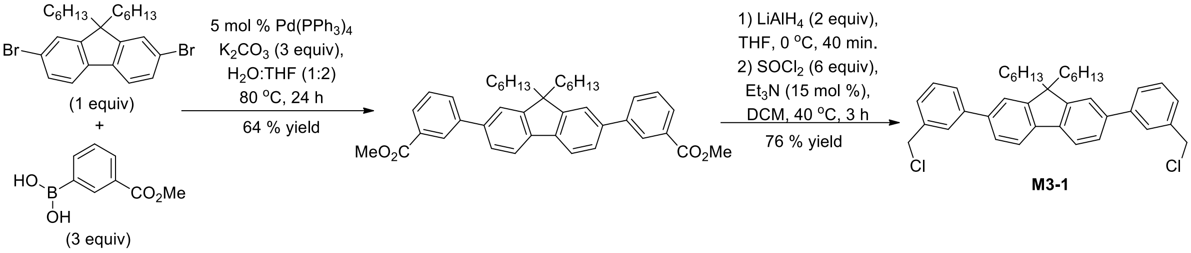

Dimethyl 3,3'-(9,9-dihexylfluorene-2,7-diyl)dibenzoate

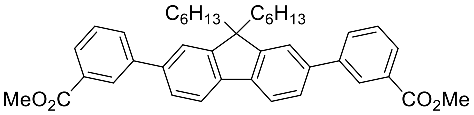

An oven-dried 250 mL three-neck round-bottom flask equipped with a stir bar was charged with 2,7-dibromo-9,9-dihexylfluorene (9.8 g, 20 mmol), 3-carboxymethylphenylboronic acid (10.8 g, 60 mmol), $Pd(PPh_3)_4$ (1.2 g, 1 mmol) and K_2CO_3 (8.3 g, 60 mmol). The round-bottom flask was equipped with a condenser (connected with circulating water). The top of the condenser were sealed by a rubber septum fitted with a balloon attached with a needle. The entire apparatus was connected to a Schlenk line, and evacuated and refilled with nitrogen (repeated three times). THF (120 mL) and H_2O (60 mL) were added under nitrogen via syringe through the rubber septum and the solution was heated to 80 °C and stirred for 24 h. After this time, the reaction mixture was cooled to room temperature and extracted with ethyl acetate (40 mL × 3). The combined organic phases was washed with brine, dried over anhydrous Na_2SO_4 and concentrated under reduced pressure. The resulting residue was purified by column chromatography (silica gel, hexanes : ethyl acetate = 20 : 1) to afford the product dimethyl 3,3'-(9,9-dihexylfluorene-2,7-diyl)dibenzoate (7.7 g, 64% yield) as a white solid.

m.p. 126-128 °C. R_f = 0.36 (hexanes : ethyl acetate = 20 : 1). ^{1}H NMR (400 MHz, $CDCl_3$) δ 8.37 (s, 2H), 8.04 (d, J = 8.0 Hz, 2H), 7.88 (d, J = 8.0 Hz, 2H), 7.81 (d, J = 8.0 Hz, 2H), 7.64 – 7.53 (m, 6H), 3.97 (s, 6H), 2.25 – 1.98 (m, 4H), 1.30 – 0.97 (m, 12H), 0.76 – 0.67 (m, 10H) ppm; ^{13}C{^{1}H} NMR (100 MHz, $CDCl_3$) δ 167.4, 152.0, 142.0, 140.6, 139.3, 131.9, 130.9, 129.1, 128.4, 128.3, 126.4, 121.6, 120.4, 55.6, 52.4, 40.6, 31.6, 29.8, 23.9, 22.7, 14.2 ppm. IR (thin film): 2928, 2856, 1717, 1468, 1435, 1281, 1253, 1111, 757 cm^{-1}; HRMS calc'd for $C_{41}H_{47}O_4^+$ 603.8095, observed 603.8093 [M+H]$^+$.

2,7-Bis(3-(chloromethyl)phenyl)-9,9-dihexylfluorene (M3-1)

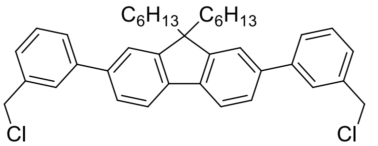

M3-1

An oven-dried 100 mL Schlenk tube equipped with a stir bar was charged with dimethyl 3,3'-(9,9-dihexylfluorene-2,7-diyl)dibenzoate (2.4 g, 4 mmol). The Schlenk tube was sealed with a rubber septum, connected to a Schlenk line, and evacuated and refilled with nitrogen (repeated three times). Anhydrous THF (15 mL) was added under nitrogen via syringe through the rubber septum, the solution was cooled to 0 ^{o}C and then was stirred at 0 ^{o}C for 30 min. Next, 4 mL of $LiAlH_4$ (8 mmol, 2.0 M solution in THF) was added dropwise into the Schlenk tube via syringe over 5 min. The mixture was stirred for 40 min under nitrogen at 0 ^{o}C. Next, 16 mL HCl solution in water (1 M) was slowly added to quench the reaction and the reaction mixture was extracted with ethyl acetate (20 mL × 3). The combined organic phases were washed with brine, dried over anhydrous Na_2SO_4, and mixture was concentrated in a 100 mL round bottom flask under reduced pressure. The product mixture was next dried under vacuum for 12 h and used as obtained in the next step.

The above mentioned 100 mL round bottom flask was next charged with a stir bar. Dichloromethane (60 mL) was added via syringe and the solution was cooled to 0 ^{o}C. Thionyl chloride (1.8 mL, 24 mmol) and triethylamine (0.08 mL, 0.6 mmol), respectively, were added dropwise into the flask via syringes over 5 min at 0 ^{o}C. After all reagents were added, the flask was equipped with a condenser (connected with circulating water). The top of the condenser were sealed by a rubber septum fitted with a balloon attached with a needle. The reaction mixture was then heated in an oil bath at 40 ^{o}C. After 5 h, the reaction mixture was cooled to room temperature. The reaction was quenched with water (20 mL), the layers were separated. The reaction mixture was extracted with dichloromethane (40 mL × 3). The combined organic phases were washed with brine, dried over anhydrous Na_2SO_4, and then concentrated under reduced pressure. The resulting residue was purified by column chromatography (silica gel, hexanes to hexanes : ethyl acetate = 20 : 1) to afford the product 2,7-bis(3-(chloromethyl)phenyl)-9,9-dihexylfluorene (**M3-1)** (1.77 g, 76% yield) as a white solid.

m.p. 99-100 ^{o}C. R_f = 0.64 (hexanes : ethyl acetate = 20 : 1). ^{1}H NMR (400 MHz, $CDCl_3$) δ 7.78 (d, J = 7.6 Hz, 2H), 7.68 (s, 2H), 7.63 (d, J = 7.6 Hz, 2H), 7.61 – 7.56 (m, 4H), 7.47 (t, J = 7.6 Hz, 2H), 7.39 (d, J = 7.6 Hz, 2H), 4.69 (s, 4H), 2.08 – 2.04 (m, 4H), 1.13 – 1.01 (m, 12H), 0.77 – 0.67 (m, 10H) ppm; $^{13}C\{^{1}H\}$ NMR (100 MHz, $CDCl_3$) δ 151.9, 142.5, 140.5, 139.7, 138.2, 129.5, 127.6, 127.5, 126.3, 121.7, 120.3, 55.6, 46.6, 40.7, 31.7, 29.9, 23.9, 22.8, 14.2 ppm. Not all resonances were observed due to overlap. IR (thin film): 2925, 2849, 1464, 1261, 823, 798, 708 cm^{-1}; HRMS calc'd for $C_{39}H_{45}Cl_2^+$ 584.6806, observed 584.6801 $[M+H]^+$.

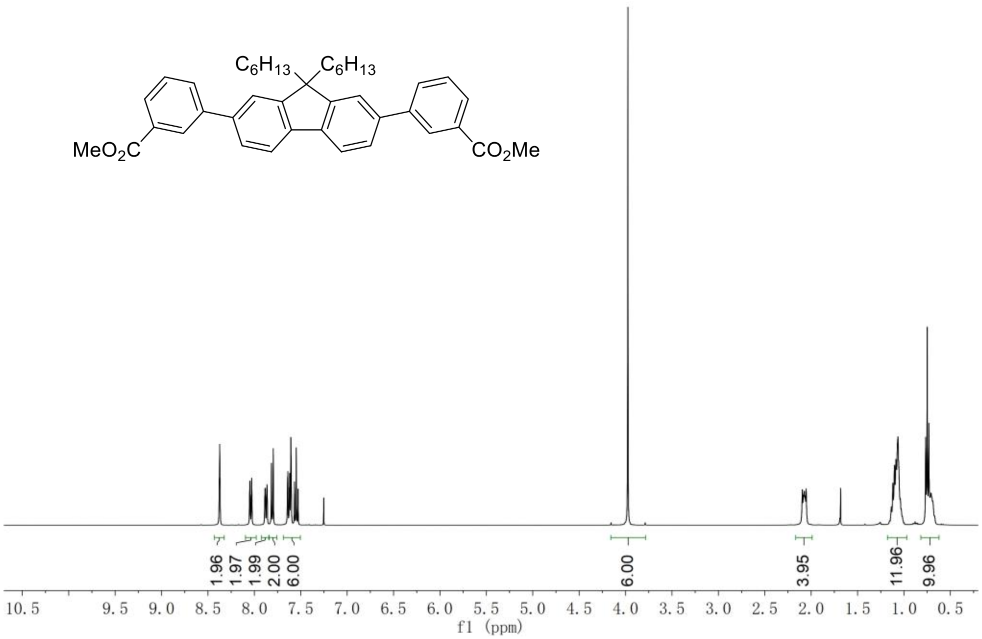

Supplementary Figure 9. Dimethyl 3,3'-(9,9-dihexylfluorene-2,7-diyl)dibenzoate (^{1}H NMR spectra, $CDCl_3$, 400 MHz)

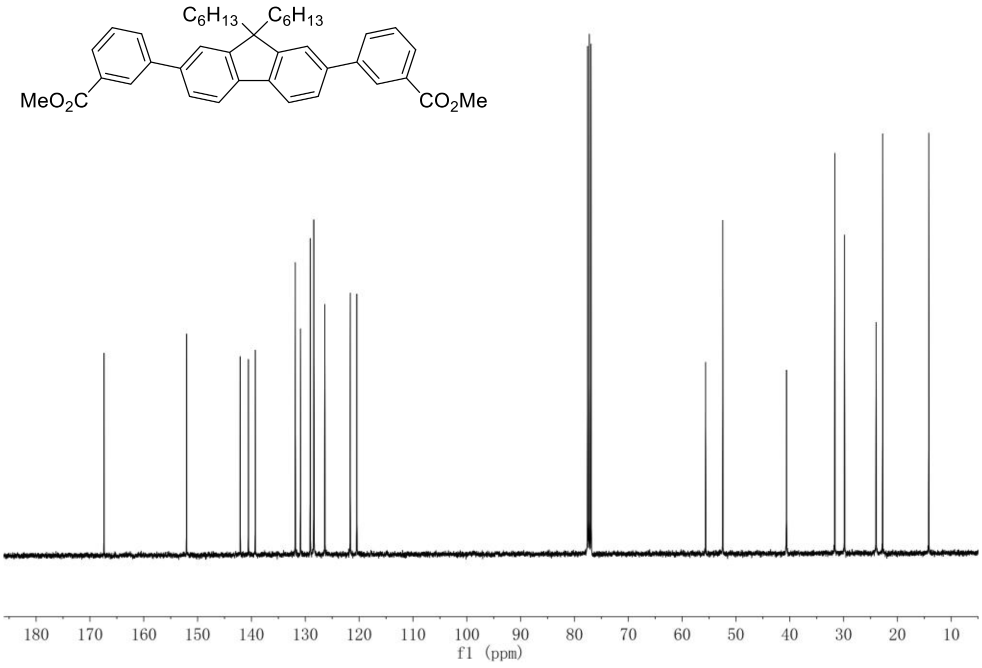

Supplementary Figure 10. Dimethyl 3,3'-(9,9-dihexylfluorene-2,7-diyl)dibenzoate ($^{13}C\{^1H\}$ NMR spectra, $CDCl_3$, 100 MHz)

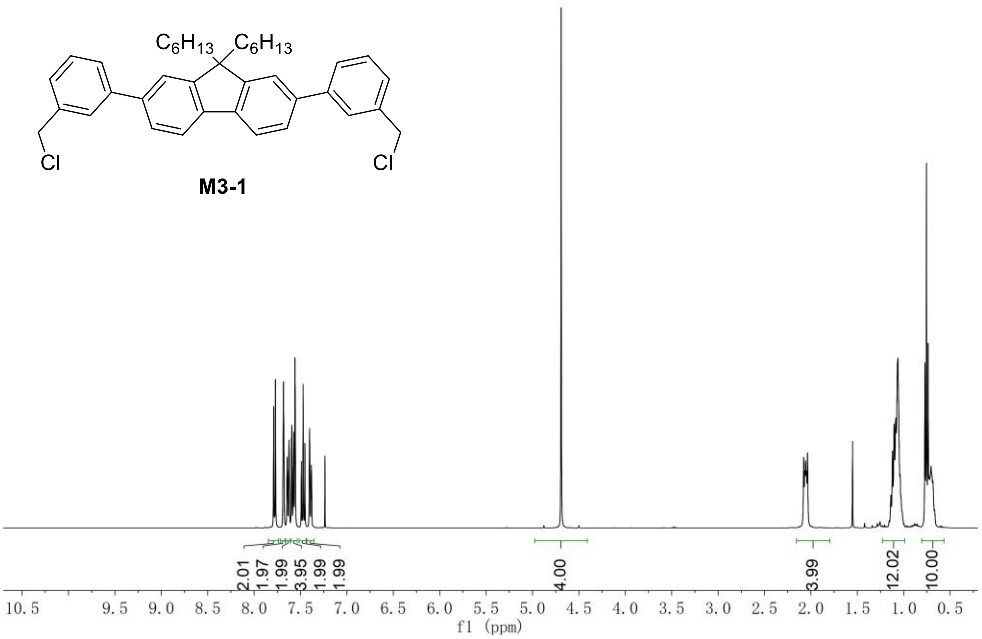

Supplementary Figure 11. 2,7-Bis(3-(chloromethyl)phenyl)-9,9-dihexylfluorene (M3-1) (^{1}H NMR spectra, $CDCl_3$, 400 MHz)

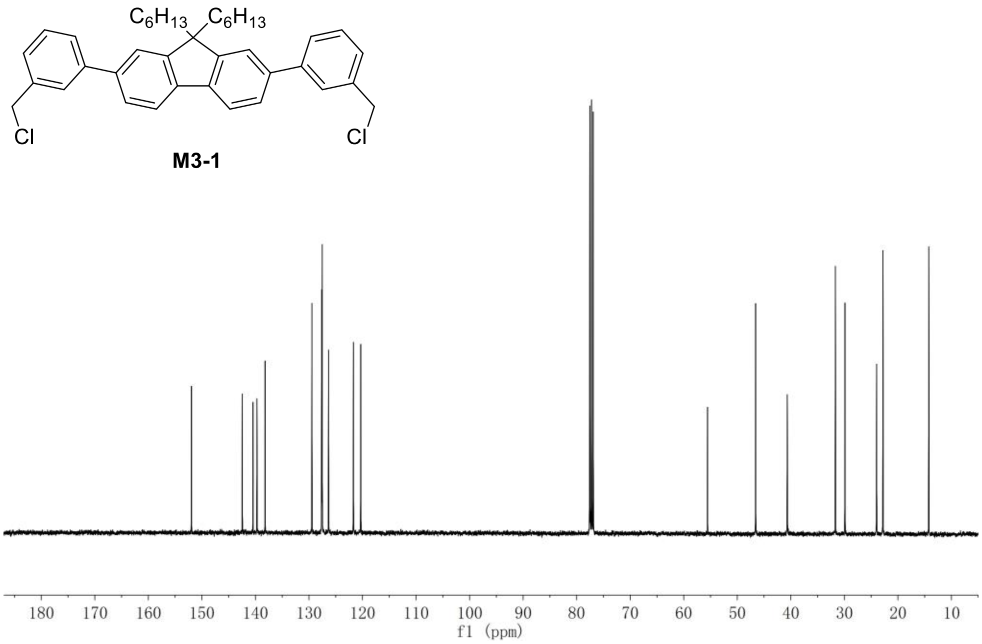

Supplementary Figure 12. 2,7-Bis(3-(chloromethyl)phenyl)-9,9-dihexylfluorene (M3-1) ($^{13}C\{^1H\}$ NMR spectra, $CDCl_3$, 100 MHz)

Preparation of monomer M4-1:

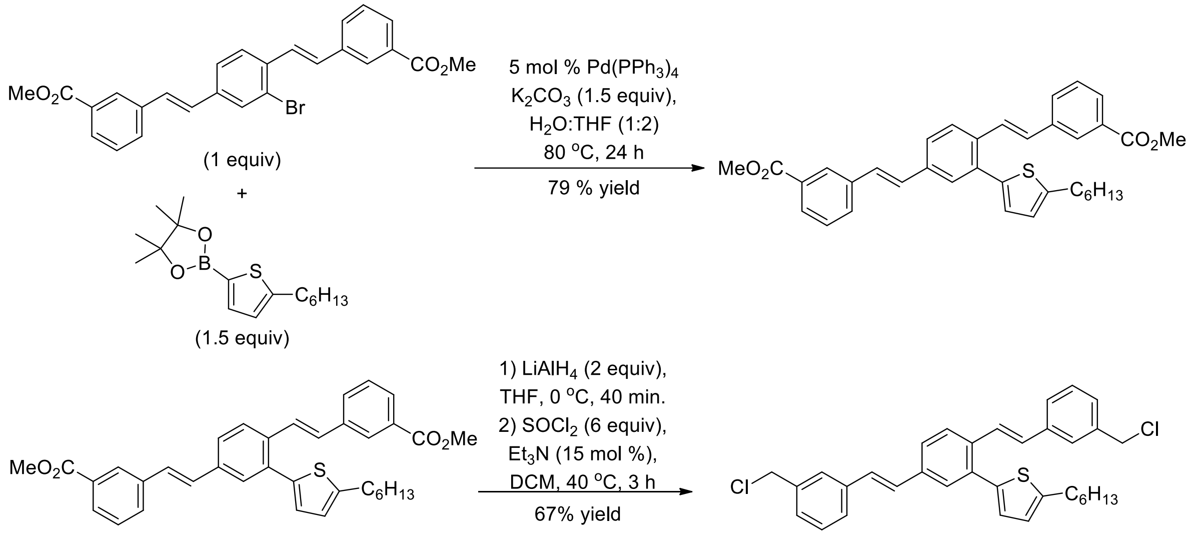

M4-1

Dimethyl 3,3'-((1E,1'E)-(2-bromo-1,4-phenylene)bis(ethene-2,1-diyl))dibenzoate:

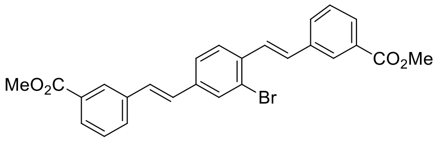

Dimethyl 3,3'-((1*E*,1'*E*)-(2-bromo-1,4-phenylene)bis(ethene-2,1-diyl))dibenzoate was prepared following literature procedure.

R_f = 0.46 (hexanes : ethyl acetate = 5 : 1). ^{1}H NMR (400 MHz, $CDCl_3$) δ 8.19 (d, *J* = 1.6 Hz, 2H), 7.96 – 7.92 (m, 2H), 7.74 – 7.72 (m, 2H), 7.67 – 7.64 (m, 2H), 7.51 (d, *J* = 16.4 Hz, 1H), 7.46 – 7.41 (m, 3H), 7.18 – 7.02 (m, 3H), 3.95 (s, 6H) ppm; ^{13}C{^{1}H} NMR (100 MHz, $CDCl_3$) δ 167.13, 167.10, 138.3, 137.5, 137.3, 136.1, 131.2, 131.1, 131.0, 130.9, 130.8, 130.4, 129.2, 129.12, 129.11, 129.04, 129.03, 128.29, 128.27, 128.0, 127.80, 126.9, 125.9, 124.8, 52.4 ppm. IR (thin film): 2950, 1724, 1436, 1292, 1196, 1106, 957, 745, 680 cm^{-1}; HRMS calc'd for $C_{26}H_{22}BrO_4^+$ 478.3545, observed 478.3552 $[M+H]^+$.

Dimethyl 3,3'-((1*E*,1'*E*)-(2-(5-hexylthiophen-2-yl)-1,4-phenylene)bis(ethene-2,1-diyl))dibenzoate:

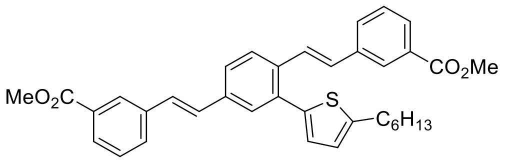

An oven-dried 250 mL three-neck round-bottom flask equipped with a stir bar was charged with dimethyl 3,3'-((1*E*,1'*E*)-(2-bromo-1,4-phenylene)bis(ethene-2,1-diyl))dibenzoate (4.8 g, 10 mmol), 2-(5-hexylthiophen-2-yl)-4,4,5,5-tetramethyl-1,3,2-dioxaborolane (4.4 g, 15 mmol), $Pd(PPh_3)_4$ (0.58 g, 0.5 mmol) and K_2CO_3 (2.0 g, 15 mmol). The round-bottom flask was equipped with a condenser (connected with circulating water). The top

of the condenser were sealed by a rubber septum fitted with a balloon attached with a needle. The entire apparatus was evacuated and refilled with nitrogen (repeated three times). THF (40 mL) and H_2O (20 mL) were added under nitrogen via syringe through the rubber septum, the solution was heated to 80 ^oC and stirred at 80 ^oC for 24 h before being cooled to room temperature. The reaction mixture was extracted with ethyl acetate (40 mL × 3). The combined organic phases was washed with brine, dried over anhydrous Na_2SO_4 and concentrated. The residue was purified by column chromatography (silica gel, hexanes : ethyl acetate = 20 : 1) to afford the product dimethyl 3,3'-((1E,1'E)-(2-(5-hexylthiophen-2-yl)-1,4-phenylene)bis(ethene-2,1-diyl))dibenzoate (4.5 g, 79% yield) as a white solid.

R_f = 0.57 (hexanes : ethyl acetate = 5 : 1). ^{1}H NMR (400 MHz, $CDCl_3$) δ 8.20 (s, 1H), 8.11 (s, 1H), 7.97 – 7.88 (m, 2H), 7.70 – 7.63 (m, 3H), 7.59 (d, J = 1.6 Hz, 1H), 7.50 – 7.38 (m, 4H), 7.17 (s, 2H), 7.07 (d, J = 16.4 Hz, 1H), 6.93 (d, J = 3.6 Hz, 1H), 6.81 (d, J = 3.6 Hz, 1H), 3.93 (d, J = 7.2 Hz, 6H), 2.87 (t, J = 7.2 Hz, 2H), 1.77 – 1.70 (m, 2H), 1.44 – 1.31 (m, 6H), 0.92 – 0.88 (m, 3H) ppm; $^{13}C\{^1H\}$ NMR (100 MHz, $CDCl_3$) δ 167.2, 147.2, 139.4, 138.1, 137.7, 136.6, 135.2, 134.6, 131.0, 130.79, 130.78, 130.73, 129.2, 129.0, 128.97, 128.91, 128.83, 128.7, 128.26, 128.19, 127.78, 127.72, 127.0, 125.8, 124.7, 52.41, 52.38, 31.86, 31.78, 30.4, 29.1, 22.7, 14.3 ppm. IR (thin film): 2928, 1723, 1438, 1290, 1201, 1108, 962, 751, 685 cm^{-1}; HRMS calc'd for $C_{36}H_{37}O_4S^+$ 565.7416, observed 565.7415 $[M+H]^+$.

2-(2,5-Bis((*E*)-3-(chloromethyl)styryl)phenyl)-5-hexylthiophene :

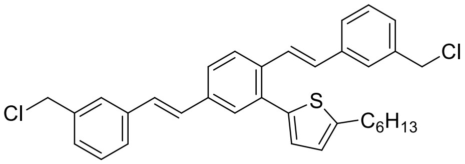

An oven-dried 100 mL Schlenk tube equipped with a stir bar was charged with dimethyl 3,3'-((1*E*,1'*E*)-(2-(5-hexylthiophen-2-yl)-1,4-phenylene)bis(ethene-2,1-diyl))dibenzoate (2.8 g, 5 mmol). The Schlenk tube was sealed with a rubber septum, connected to a Schlenk line, and evacuated and refilled with nitrogen (repeated three times). Anhydrous THF (20 mL) was added under nitrogen via syringe through the rubber septum, the solution was cooled to 0 ^oC, and the reaction mixture was stirred at 0 ^oC for 30 min. Next, 5 mL of $LiAlH_4$ (10 mmol, 2.0 M solution in THF) was added dropwise into the Schlenk tube via syringe over 5 min. The mixture was stirred for 40 min under nitrogen at 0 ^oC. 18 mL 1 M HCl was slowly added to quench the reaction and then the reaction mixture was extracted with ethyl acetate (30 mL × 3). The combined organic phases were washed with brine, dried over anhydrous Na_2SO_4, and mixture was concentrated in a 100 mL round bottom flask. The mixture was next dried under vacuum for 12 h and the resulting compound used as obtained in the next step.

The above mentioned 100 mL round bottom flask was charged with a stir bar. Dichloromethane (60 mL) was added via syringe and the solution was cooled to 0 ^oC. Thionyl chloride (1.8 mL, 24 mmol) was added dropwise into the flask over 5 min at 0 ^oC followed by triethylamine (0.08 mL, 0.6 mmol) over 5 min at 0 ^oC

both via syringe. After all reagents were added, the flask was equipped with a condenser (connected with circulating water). The top of the condenser were sealed by a rubber septum fitted with a balloon attached with a needle. The reaction mixture was then heated in an oil bath at 40 ºC. After 5 h, the reaction mixture was cooled to room temperature and was quenched with water (20 mL), the layers were separated and the reaction mixture was extracted with dichloromethane (40 mL × 3). The combined organic phases were washed with brine, dried over anhydrous Na_2SO_4, and then concentrated under reduced pressure. The residue was purified by column chromatography (silica gel, hexanes to hexanes : ethyl acetate = 20 : 1) to afford the product 2-(2,5-bis((*E*)-3-(chloromethyl)styryl)phenyl)-5-hexylthiophene (**M4-1)** (1.46 g, 67% yield) as a brown thick oil.

R_f = 0.54 (hexanes : ethyl acetate = 20 : 1). ^{1}H NMR (400 MHz, $CDCl_3$) δ 7.67 (d, *J* = 8.0 Hz, 1H), 7.61 – 7.50 (m, 2H), 7.50 – 7.38 (m, 5H), 7.37 – 7.24 (m, 4H), 7.12 (s, 2H), 7.02 (d, *J* = 16.4 Hz, 1H), 6.92 (d, *J* = 3.5 Hz, 1H), 6.79 (d, *J* = 3.5 Hz, 1H), 4.58 (d, *J* = 8.4 Hz, 4H), 2.86 (t, *J* = 8.4 Hz, 2H), 1.82 – 1.65 (m, 2H), 1.42 – 1.30 (m, 6H), 0.97 – 0.83 (m, 3H) ppm; ^{13}C{^{1}H} NMR (100 MHz, $CDCl_3$) δ 147.2, 139.5, 138.4, 138.09, 138.06, 137.9, 136.6, 135.2, 134.5, 129.31, 129.29, 129.27, 128.9, 128.8, 128.6, 128.5, 128.0, 127.8, 127.7, 127.1, 126.9, 126.8, 126.7, 126.6, 125.7, 124.7, 46.38, 31.89, 31.79, 30.42, 29.08, 22.80, 14.32, 46.38, 31.89, 31.79, 30.4, 29.1, 22.8, 14.3 ppm. IR (thin film): 2927, 2854, 1601, 1493, 1264, 960, 813, 738, 706 cm^{-1}; HRMS calc'd for $C_{34}H_{35}Cl_2S^+$ 546.6127, observed 546.6122 $[M+H]^+$.

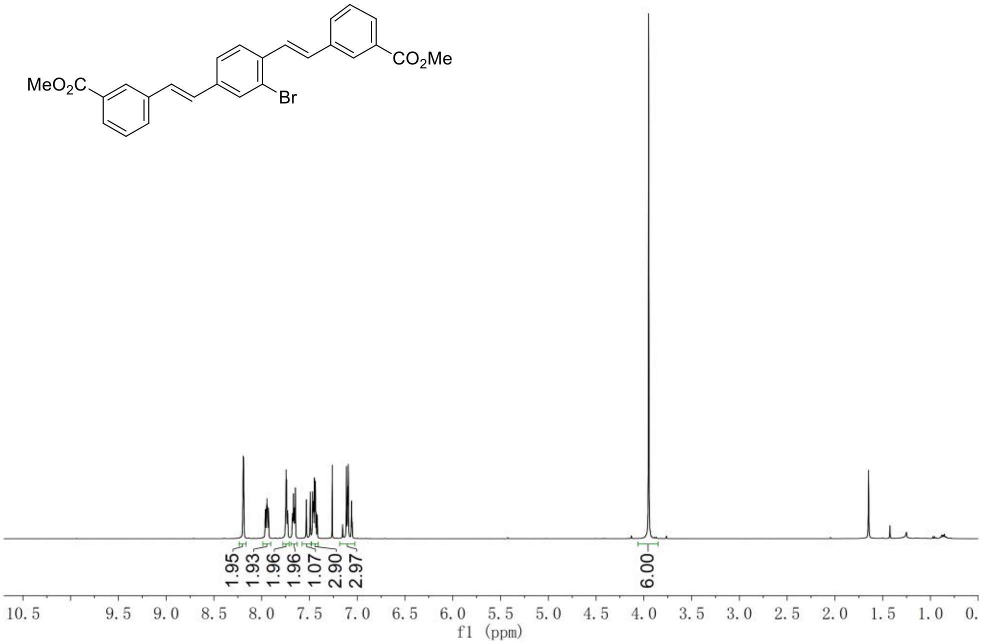

Supplementary Figure 13.

Dimethyl 3,3'-((1E,1'E)-(2-bromo-1,4-phenylene)bis(ethene-2,1-diyl))dibenzoate:

(^{1}H NMR spectra, $CDCl_3$, 400 MHz)

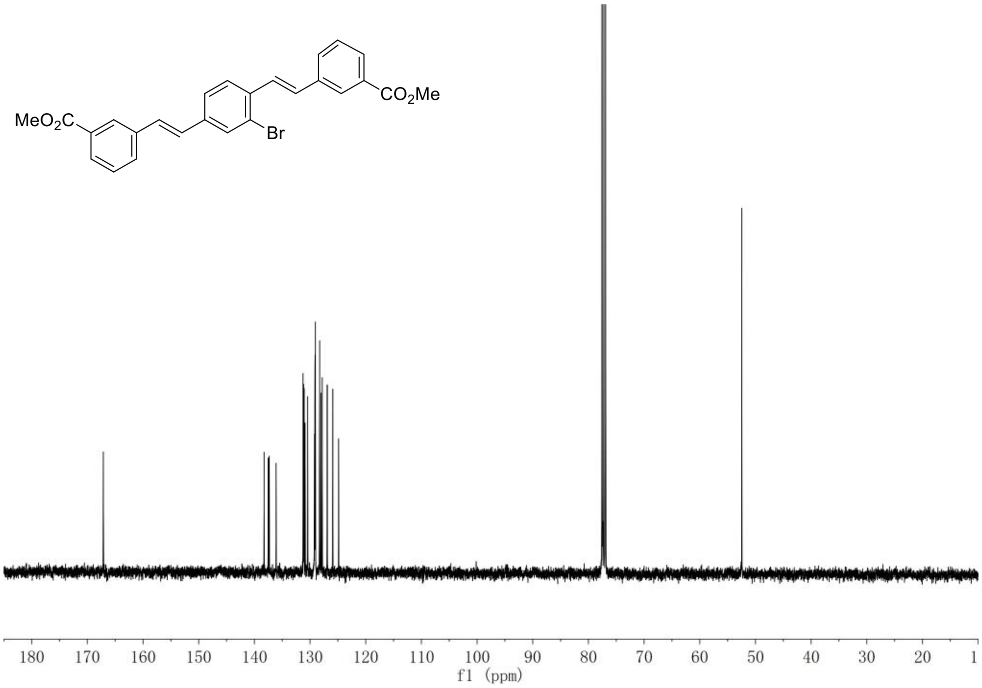

Supplementary Figure 14.

Dimethyl 3,3'-((1E,1'E)-(2-bromo-1,4-phenylene)bis(ethene-2,1-diyl))dibenzoate:

($^{13}C\{^{1}H\}$ NMR spectra, $CDCl_3$, 100 MHz)

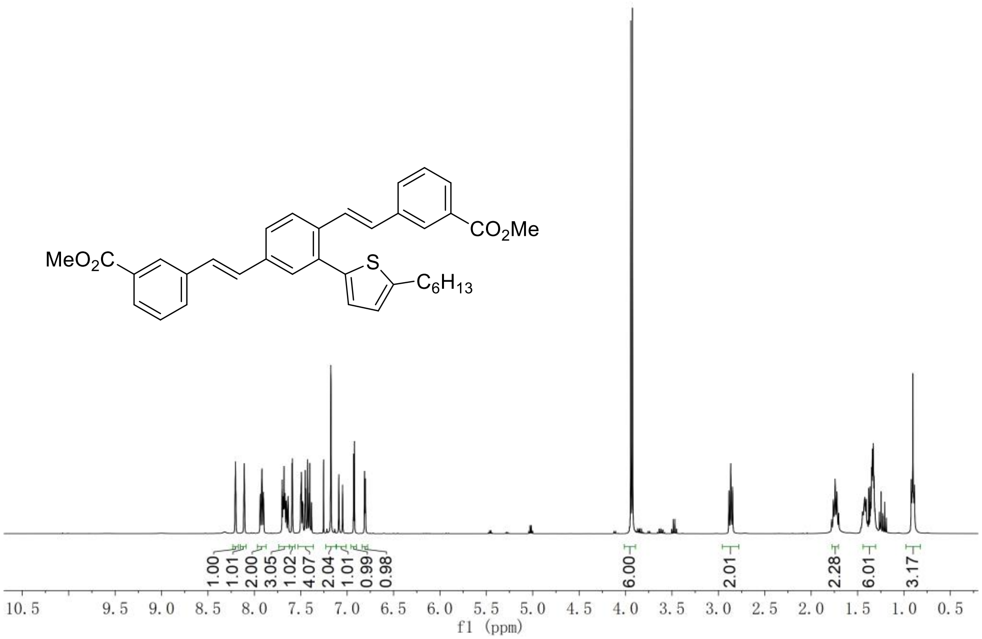

Supplementary Figure 15.

Dimethyl 3,3'-((1E,1'E)-(2-(5-hexylthiophen-2-yl)-1,4-phenylene)bis(ethene-2,1-diyl))dibenzoate

(^{1}H NMR spectra, $CDCl_3$, 400 MHz)

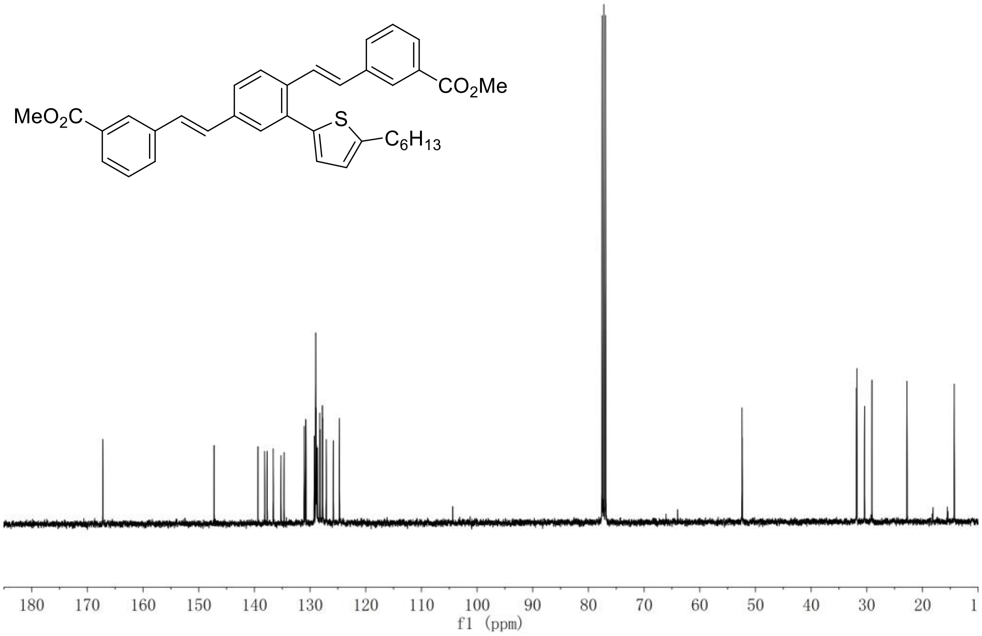

Supplementary Figure 16.

Dimethyl 3,3'-((1E,1'E)-(2-(5-hexylthiophen-2-yl)-1,4-phenylene)bis(ethene-2,1-diyl))dibenzoate

($^{13}C\{^{1}H\}$ NMR spectra, $CDCl_3$, 100 MHz)

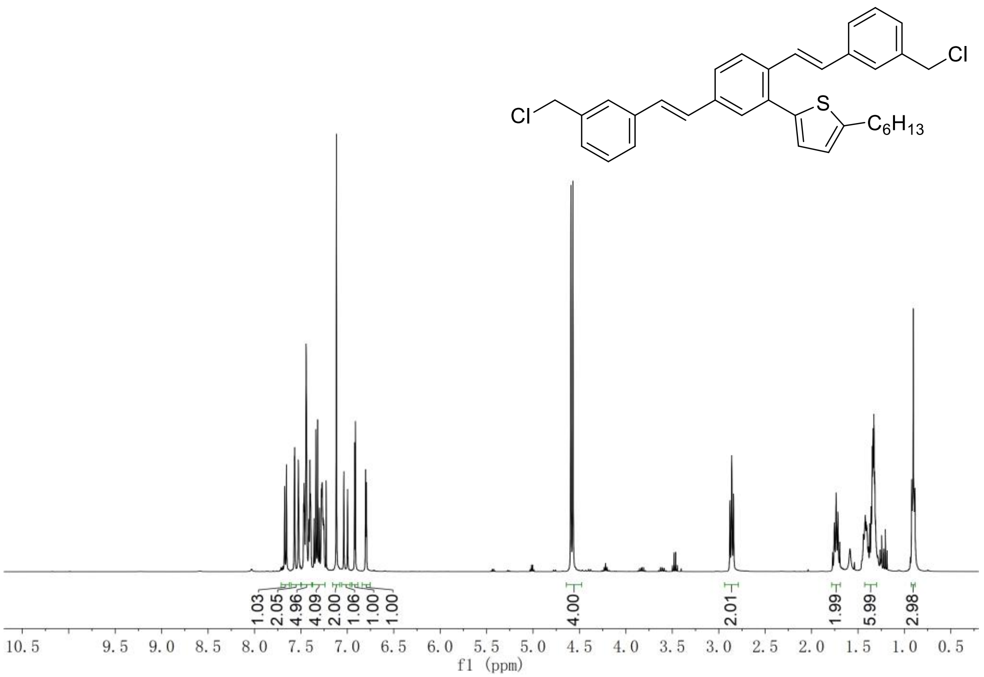

Supplementary Figure 17.

2-(2,5-Bis((E)-3-(chloromethyl)styryl)phenyl)-5-hexylthiophene

(^{1}H NMR spectra, $CDCl_3$, 400 MHz)

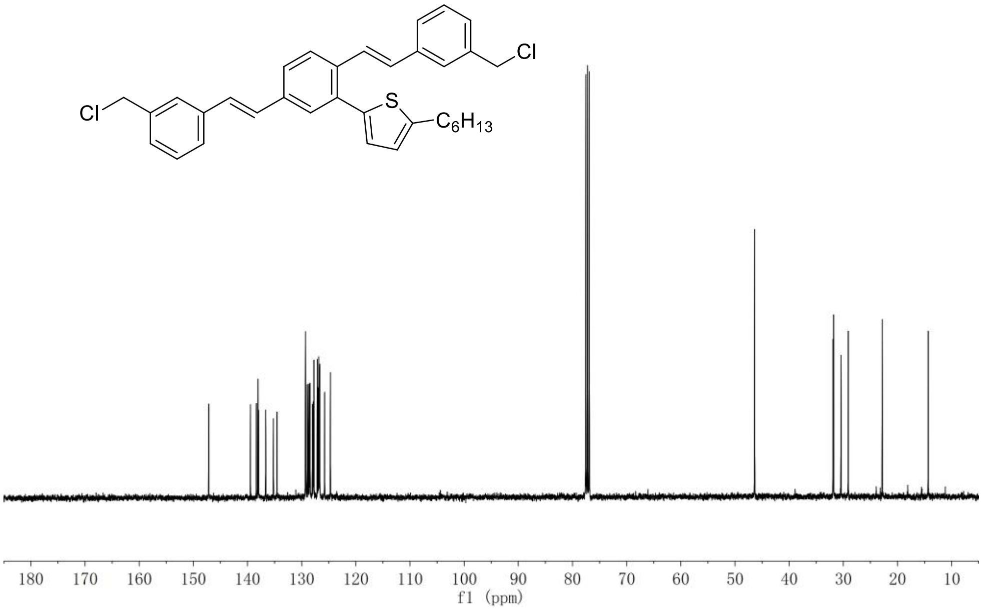

Supplementary Figure 18.

2-(2,5-Bis((E)-3-(chloromethyl)styryl)phenyl)-5-hexylthiophene

($^{13}C\{^{1}H\}$ NMR spectra, $CDCl_3$, 100 MHz)

High-Throughput Experimentation screenings for polymerization:

Parallel High-throughput Experimentation Screening was accomplished in an MBraun glovebox operating with a constant N_2-purge (oxygen typically <5 ppm). The experimental design was accomplished using Accelrys Library Studio. Screening reactions were carried out in 1 mL vials (30 mm height×8 mm diameter) in 24-well plate aluminum reactor block. Liquid chemicals were dosed using multi-channel or single-channel pipettors. Solid chemicals were dosed manually as solutions or slurries in appropriate solvents. Undesired additional solvent was removed using a GeneVac system located inside the glovebox. The reactions were heated and stirred on a heating block with a tumble-stirrer (V&P Scientific) using 1.98 mm diameter×4.80 mm length parylene stir bars. The tumble stirring mechanism helped to insure uniform stirring throughout the 96-well plate. The reactions were sealed in the 96-well plate during reaction. Below each reactor vial in the aluminum 96-well plate was a 0.062 mm thick silicon-rubber gasket. Directly above the glass vial reactor tops was a Teflon perfluoroalkoxy copolymer resin sealing gasket and above that, two more 0.062 mm thick silicon-rubber gaskets. The entire assembly was compressed between an aluminum top and the reactor base with 9 evenly-placed screws.

General procedure

Set up:

Experiments were set up inside a glovebox under a nitrogen atmosphere. A 96-well aluminum block containing 1 mL glass vials was dosed with sulfoxide catalyst in THF. The solvent was removed to dryness using a GeneVac. Monomer **1** (10 μmol) and the corresponding base (30 μmol) were separately dosed into each reaction vial with the corresponding solvent (100 µL each, total volume 200 µL , 0.05 M). The plate was then sealed with screwdriver and stirred for 24 h at 80 ^oC.

work up:

Upon cooling to room temperature, the plate was opened in the glovebox, water (10 µL) was added into each vial with a pipetman to quench the reactions and then solvent was removed to dryness using a GeneVac. Next, $CHCl_3$ (200 µL) was added into each vial and the slurry solution was allowed to stir for 10 min. Cold methanol (600 µL) was added into each vial to precipitate the polymer and the slurry solution was allowed to stir for 10 min. The slurry is then transferred with a multichannel pipetman onto a filter plate positioned on the vacuum slot of a Freeslate CM2 reaction deck. After the $MeOH/CHCl_3$ solution was filtered, the polymer remains on the filter plate. Finally, a 96-well collection plate was put beneath the filter plate and 800 µL THF was added into the filter plate well to dissolve the polymer. The polymer solution was transferred into a 96-well LC-block and analyzed by GPC.

Supplementary Figure 19. Work-flow of High-Throughput-Screening in polymerization study.

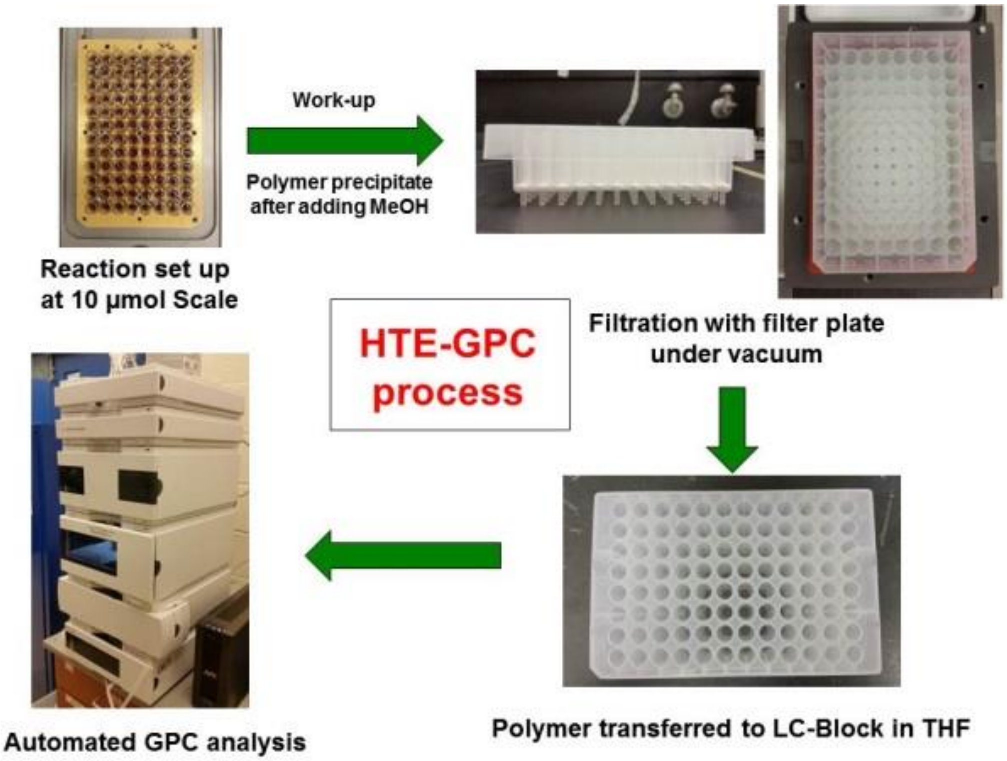

Supplementary Table 1. Screening of sulfoxide catalysts and solvents.

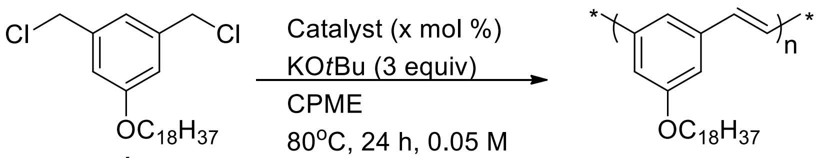

1

Variants:

Catalyst:

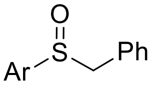

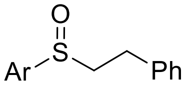

Cat.1: Ar = Ph
Cat.2: Ar = 4-C_6H_4-OMe
Cat.3: Ar = 4-C_6H_4-F
Cat.4: Ar = 4-C_6H_4-CF_3

Cat.5: Ar = 1-Naph
Cat.6: Ar = 2-Naph
Cat.7: Ar = 2-C_6H_4-Me
Cat.8: Ar = 3-Pyridyl
Cat.9: Ar = 4-Pyridyl

Cat.10

Loading: x = 10, 7.5, 5, 2.5

Entry	Loading (mol %)	M_n	PDI
Cat.1	10	11685	1.22
Cat.2	10	10755	1.25

Cat.3	10	10928	1.24
Cat.4	10	10555	1.25
Cat.5	10	10470	1.24
Cat.6	10	11260	1.26
Cat.7	10	10844	1.25
Cat.8	10	11447	1.28
Cat.9	10	10061	1.26
Cat.10	10	11761	1.23
Cat.1	7.5	11502	1.27
Cat.2	7.5	11253	1.24
Cat.3	7.5	11420	1.28
Cat.4	7.5	10582	1.25
Cat.5	7.5	10970	1.23
Cat.6	7.5	11169	1.28
Cat.7	7.5	11262	1.25
Cat.8	7.5	11272	1.26
Cat.9	7.5	8401	1.33
Cat.10	7.5	10854	1.26
Cat.1	5	10160	1.24
Cat.2	5	9960	1.25
Cat.3	5	9834	1.25
Cat.4	5	9504	1.26
Cat.5	5	7739	1.38
Cat.6	5	10253	1.25
Cat.7	5	9325	1.25
Cat.8	5	8293	1.36
Cat.9	5	5749	1.37
Cat.10	5	9638	1.27

Note: Catalyst loading to 2.5 mol % led to incomplete polymerization and data were not further processed.

Supplementary Table 2. Screening of Base.

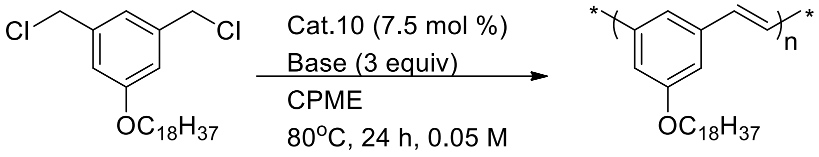

Variants: 10 bases

Base	Mn	PDI
LiO*t*Bu	No Polymerization	
NaOtBu	No Polymerization	
$LiN(SiMe_3)_2$	5500	1.35
$NaN(SiMe_3)_2$	10173	1.25
$KN(SiMe_3)_2$	8278	1.37
NaH	No Polymerization	
KH	No Polymerization	
$KOSiMe_3$	No Polymerization	
KOPh	No Polymerization	
NaOMe	No Polymerization	

Supplementary Table 3. Screening of solvent.

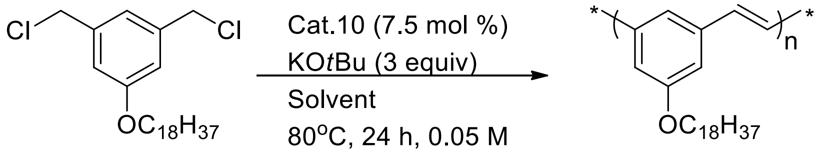

Variants: 5 solvent

Solvent	Mn	PDI
THF	10216	1.20
Dioxane	10730	1.23
MTBE	13164	1.23
Tol	9829	1.24
DMF	6271	1.55

General procedure for the 0.1 mmol lab-scale BCCP.

An oven-dried 8 mL microwave vial equipped with a stir bar was charged with monomer **M1** (44.4 mg, 0.10 mmol) under a nitrogen atmosphere in a glove box. A solution of precatalyst **10** (1.73 mg, 0.075 mmol) in 1.0 mL anhydrous CPME was added by syringe. Next, a solution of KO*t*Bu (33.6 mg, 0.30 mmol) in 1.0 mL anhydrous CPME was added by syringe. The reaction was stirred for 24 h at 80 ºC, quenched with 2 drops of H_2O via syringe, cooled to room temperature and opened to air. After the volatile materials were removed with rotary evaporator, $CHCl_3$ (2 mL) was added into each vial and the slurry solution was allowed to stir for 10 min. Cold methanol (6 mL) and H_2O (0.5 mL) was then added into each vial to precipitate the polymer and the slurry solution with polymer suspension was allowed to stir for 10 min. The mixture was then transferred with a pipette onto a Whatman autovial syringeless filter (5 mL, 0.45 µm PTFE membrane). After the MeOH/$CHCl_3$/H_2O solution was filtered, polymer that remained in the filter was washed sequentially with 5 mL MeOH and 5 mL pentane. Finally, the polymer remaining in the filter was transferred into a 20 mL vial with spatula and dried in under vacuum to yield a pale yellow solid in 33.8 mg, 91% yield.

GPC distribution plots of polymer P1 of lab-scale optimization (0.1 mmol)

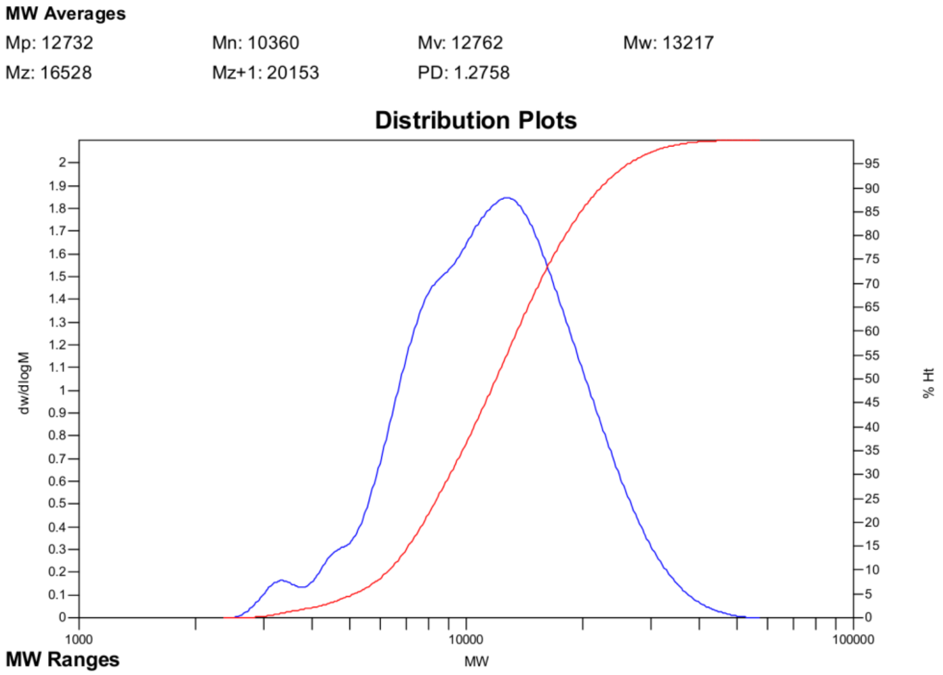

Supplementary Figure 20. GPC distribution plots of polymer P1 in Table 1, entry 1.

MW Averages

Mp: 15708 Mn: 13561 Mv: 16002 Mw: 16445

Mz: 19548 Mz+1: 22772 PD: 1.2127

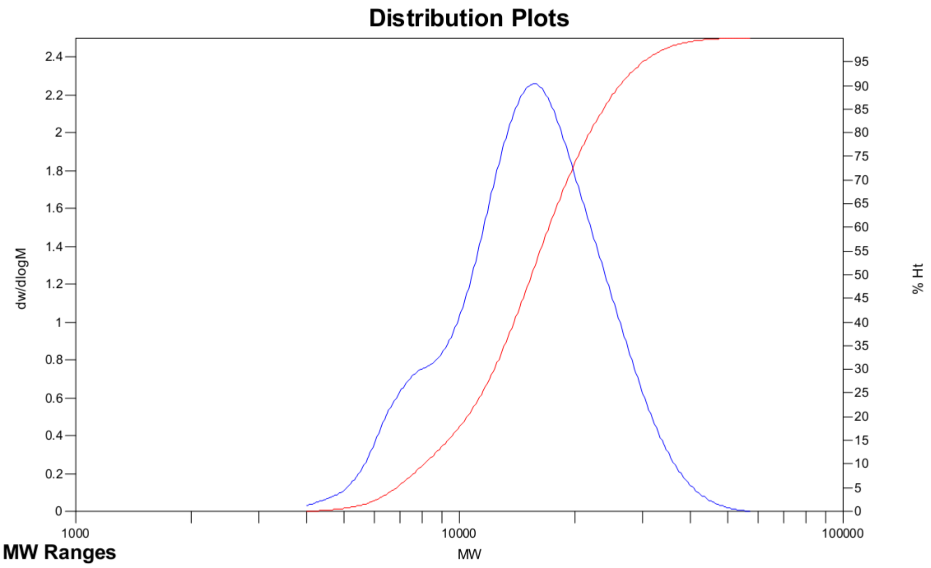

Supplementary Figure 21. GPC distribution plots of polymer P1 in Table 1, entry 2.

MW Averages

Mp: 13167 Mn: 11152 Mv: 13628 Mw: 14081

Mz: 17317 Mz+1: 20774 PD: 1.2626

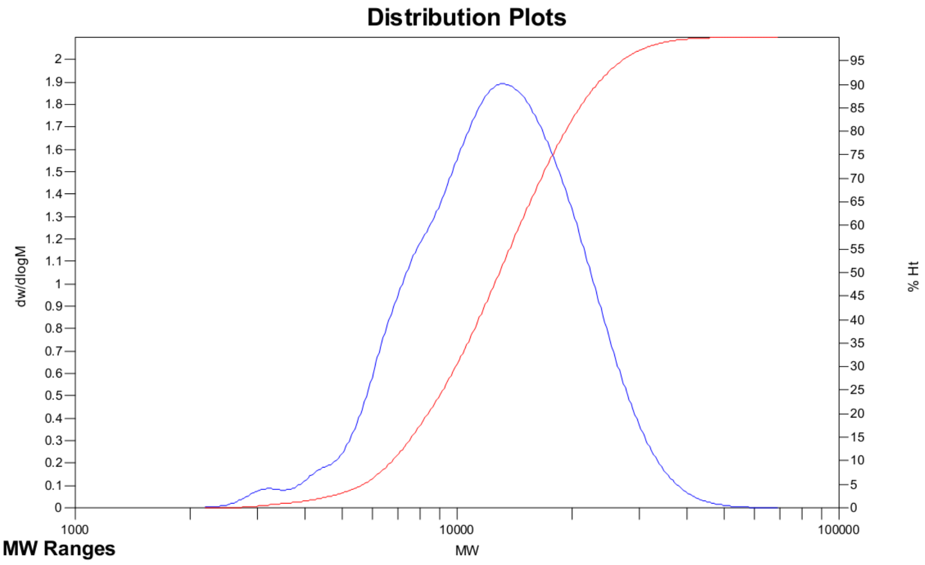

Supplementary Figure 22. GPC distribution plots of polymer P1 in Table 1, entry 3.

MW Averages

Mp: 14693	Mn: 12173	Mv: 15145	Mw: 15691
Mz: 19615	Mz+1: 23887	PD: 1.2890	

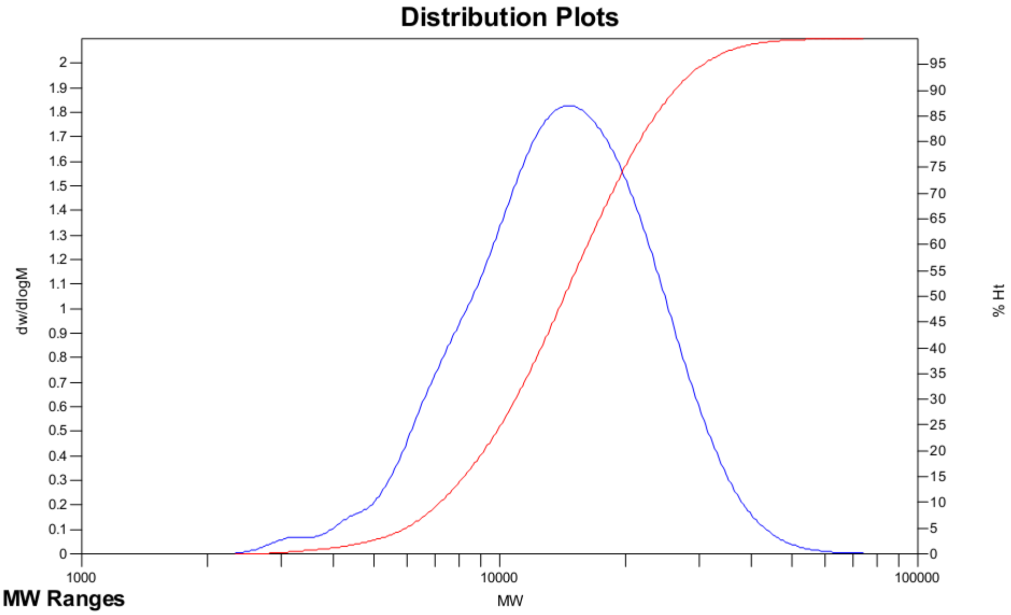

MW Ranges

Supplementary Figure 23. GPC distribution plots of polymer P1 in Table 1, entry 4.

MW Averages

Mp: 18638	Mn: 15227	Mv: 20484	Mw: 21500
Mz: 29255	Mz+1: 38526	PD: 1.4120	

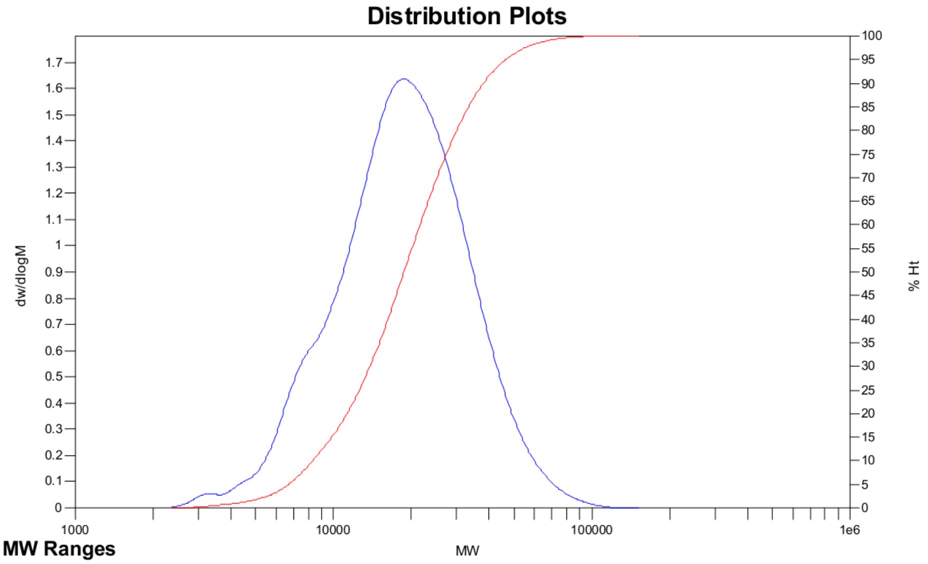

MW Ranges

Supplementary Figure 24. GPC distribution plots of polymer P1 in Table 1, entry 5.

MW Averages

Mp: 20342	Mn: 17382	Mv: 23520	Mw: 24780
Mz: 35047	Mz+1: 49323	PD: 1.4256	

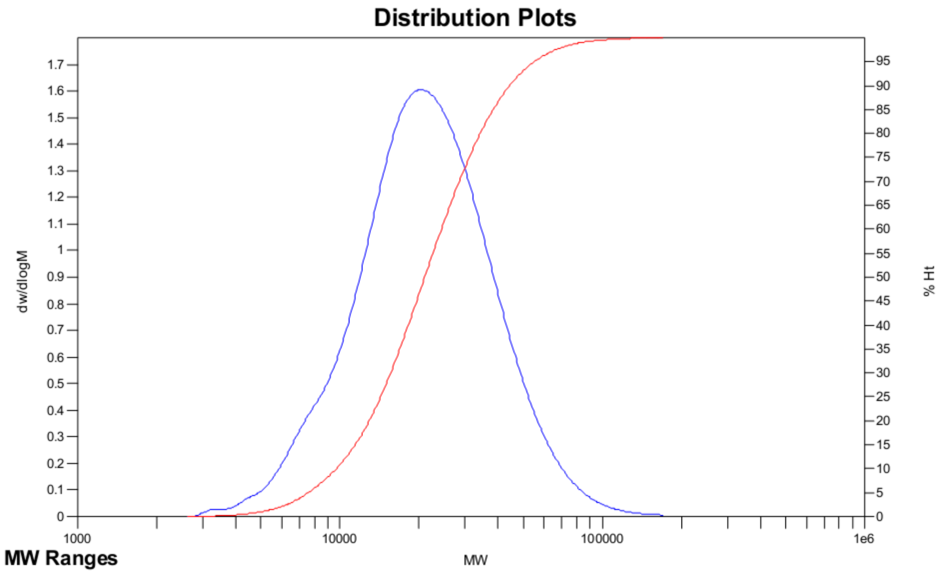

Supplementary Figure 25. GPC distribution plots of polymer P1 in Table 1, entry 6.

MW Averages

Mp: 12256	Mn: 10564	Mv: 12501	Mw: 12876
Mz: 15694	Mz+1: 19234	PD: 1.2189	

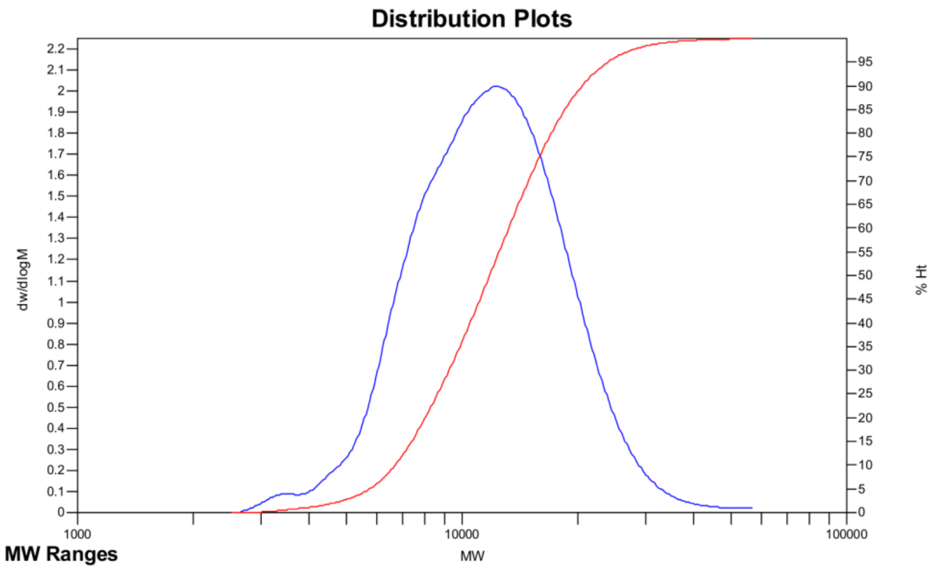

Supplementary Figure 25. GPC distribution plots of polymer P1 in Table 1, entry 7.

MW Averages

Mp: 12593	Mn: 10344	Mv: 12240	Mw: 12585
Mz: 15008	Mz+1: 17524	PD: 1.2166	

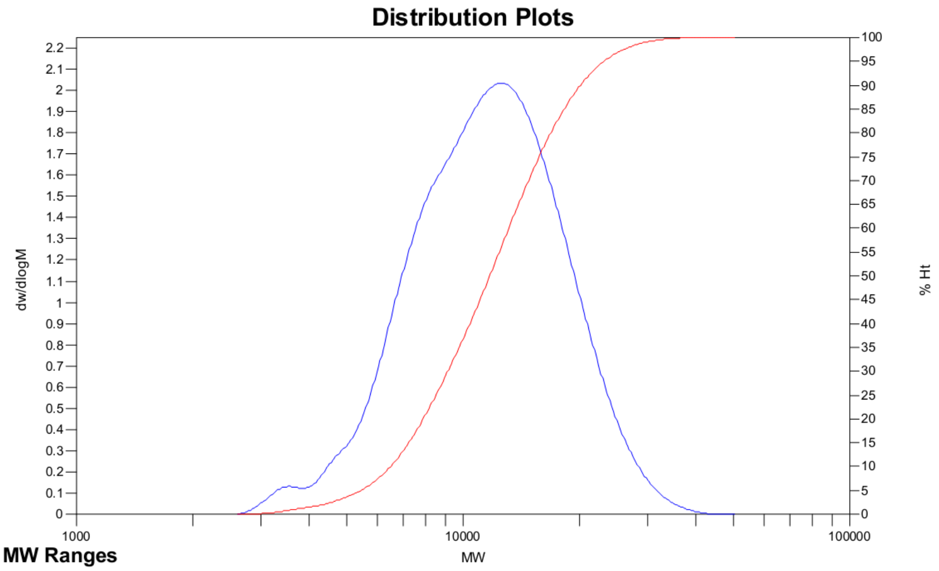

MW Ranges

Supplementary Figure 26. GPC distribution plots of polymer P1 in Table 1, entry 8.

MW Averages

Mp: 12443	Mn: 10129	Mv: 12017	Mw: 12358
Mz: 14747	Mz+1: 17208	PD: 1.2201	

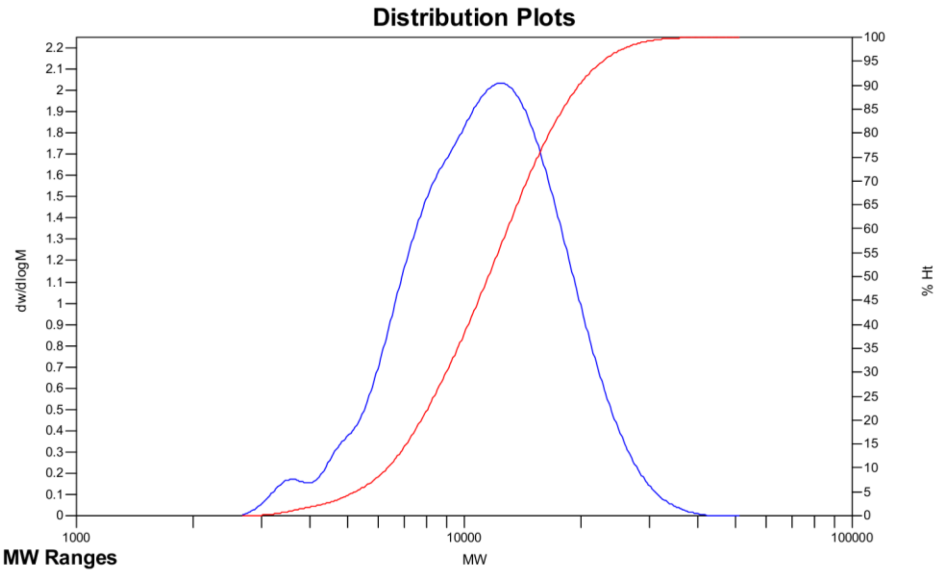

MW Ranges

Supplementary Figure 27. GPC distribution plots of polymer P1 in Table 1, entry 9.

General procedure for the scale-up (1 mmol) polymerization.

An oven-dried 100 mL Schlenk tube equipped with a stir bar was charged with monomer 1 (444.0 mg, 1.0 mmol) and precatalyst **10** (17.3 mg, 0.75 mmol). The Schlenk tube was sealed with a rubber septum and was connected to a Schlenk line, evacuated, and refilled with nitrogen (repeated three times). Next, a solution of KO*t*Bu (336 mg, 3.0 mmol) in 20 mL anhydrous CPME was added by syringe. The reaction was stirred for 24 h at 80 ºC, cooled to room temperature, opened to air and quenched with 1 mL of H_2O. The reaction mixture was firstly transferred to a 250 mL round bottom flask and the volatile materials were removed with rotary evaporator. Next, $CHCl_3$ (20 mL) was added into flask and the slurry solution was allowed to stir for 10 min. Cold methanol (60 mL) was added into the flask to precipitate the polymer and the slurry solution with polymer suspension was allowed to stir for 10 min. The mixture was then filtered on a glass fritted filter funnel (75 mL), After the MeOH/$CHCl_3$ solution was filtered, solid was washed by H_2O (5 mL), MeOH (20 mL *3), pentane (5 mL), collected and dried in a vacuum as pale yellow solid to provide 334.8 mg, 90% yield of the polymer.

MW Averages

Mp: 13132	Mn: 10595	Mv: 12367	Mw: 12668
Mz: 14695	Mz+1: 16649	PD: 1.1957	

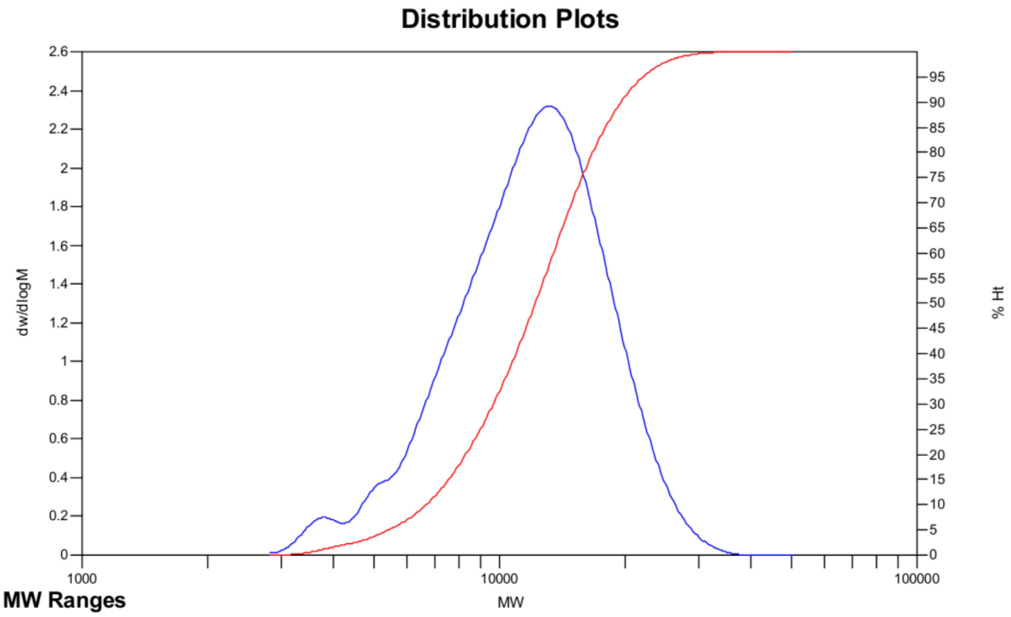

Supplementary Figure 28. GPC distribution plots of polymer P1 in 1 mmol scale synthesis.

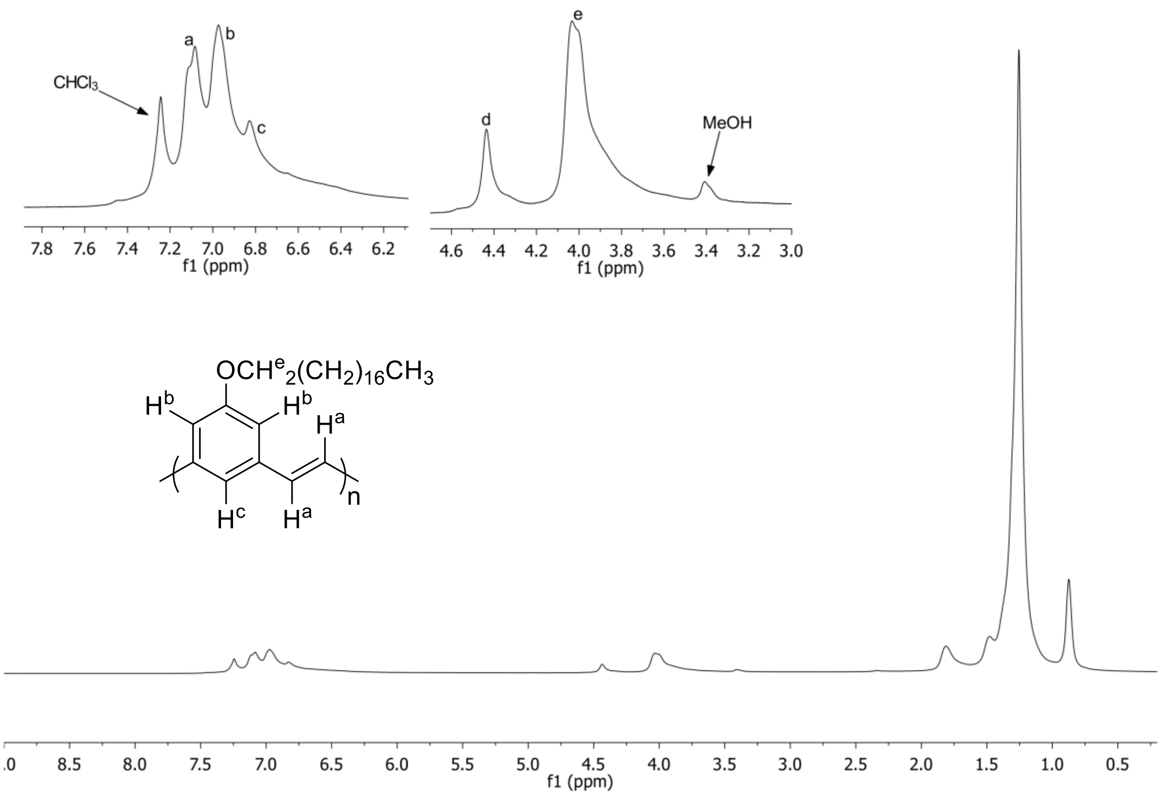

Supplementary Figure 29. ^{1}H NMR spectra of Polymer P1 (^{1}H NMR, $CDCl_3$, 500 MHz).

Discussion:

H^a at 7.11 ppm is assigned as vinyl C-H, H^b, H^c at 6.93 ppm to 6.97 ppm are assigned as phenyl C-H, H^d at 4.44 ppm is assigned as benzyl C-H on the polymer end.

H^e at 4.03 ppm is assigned as OCH_2 on the phenyl ring. Cis/trans vinylene linkage could be differentiated by NMR in model compound study reported by Fumiyuki et. al. (Wakioka, M., Ikegami, M. & Ozawa, F. Stereocontrolled Synthesis and Photoisomerization Behavior of All-Cis and All-Trans Poly(m-phenylenevinylene)s. Macromolecules **2010**, *43*, 6980-6985) in which OCH_2 on the phenyl ring in *trans*-vinylene motif is expected to be around 4.05 ppm (compound ***E,E*-3** in the literature), while OCH_2 on the phenyl ring in *trans*-vinylene motif is expected to be around 3.59 ppm (compound ***Z,Z*-3** in the literature).

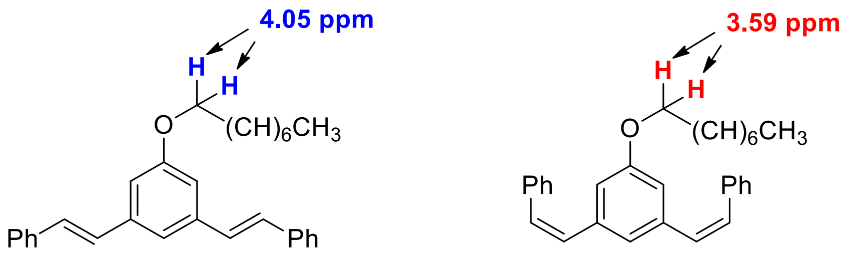

MW Averages

Mp: 17304	Mn: 14343	Mv: 19703	Mw: 20926
Mz: 32847	Mz+1: 58387	PD: 1.4590	

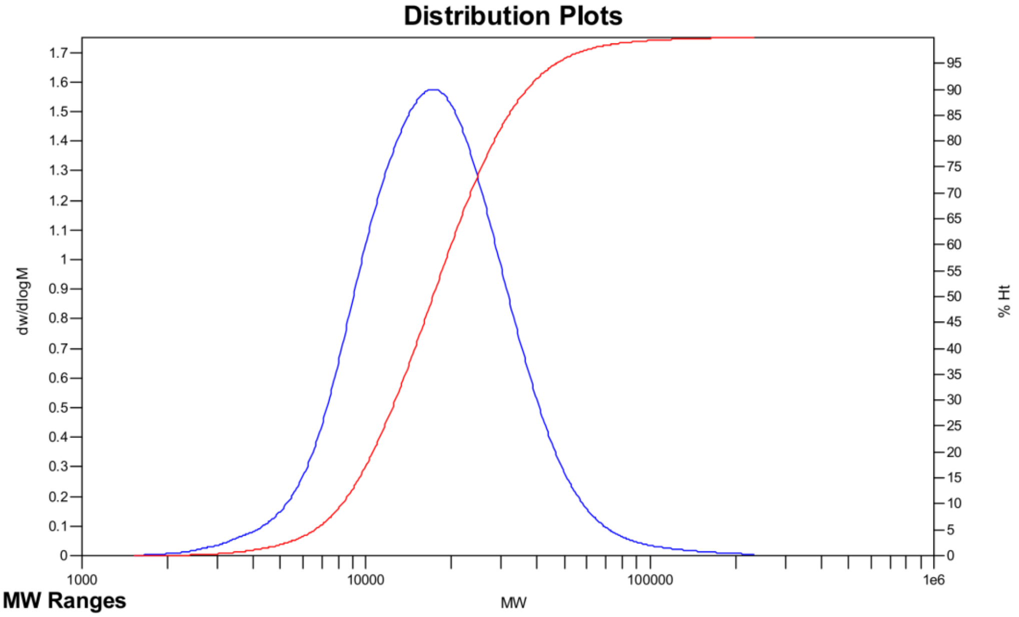

MW Ranges

Supplementary Figure 30. GPC distribution plots of polymer P2.

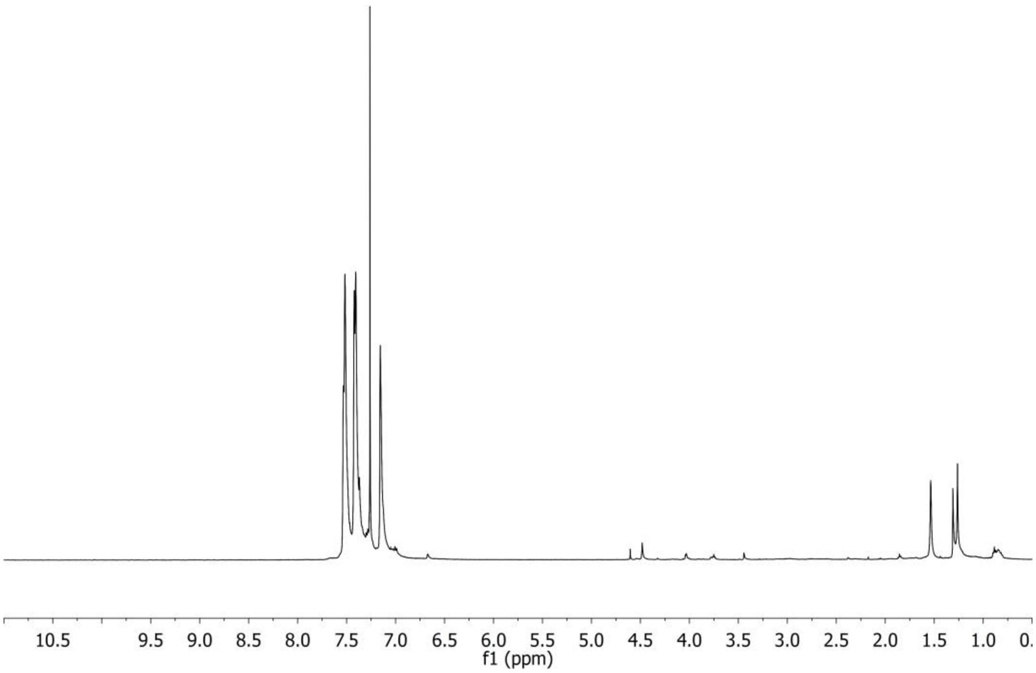

Supplementary Figure 31. ^{1}**H NMR spectra of Polymer P2 (**^{1}**H NMR, $CDCl_3$, 500 MHz).**

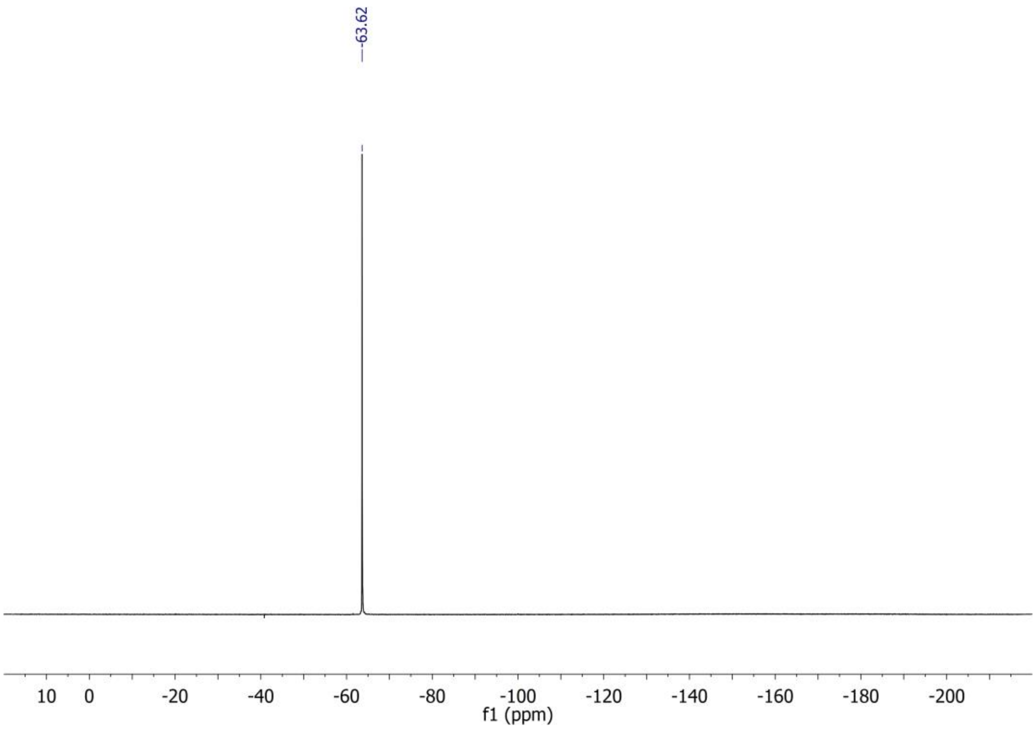

Supplementary Figure 32. ^{19}**F NMR spectra of Polymer P2 (**^{19}**F NMR, $CDCl_3$, 340 MHz).**

1 mmol Scale synthesis and characterization of co-polymer P3-1, P4-1.

Synthesis of P3-1.

Co-polymer **P3-1** was synthesized at 1 mmol scale following the general procedure of the 1 mmol scale BCCP with monomer **M3-1** (583.7 mg, 1 mmol), 1-methoxy-4-(phenethylsulfinyl)benzene (26 mg, 0.1 mmol) and KO*t*Bu (336 mg, 3.0 mmol) at 0.1 M concentration. Out of the reaction, co-polymer **P3-1** was obtained as pale yellow solid in 460 mg, 91% yield.

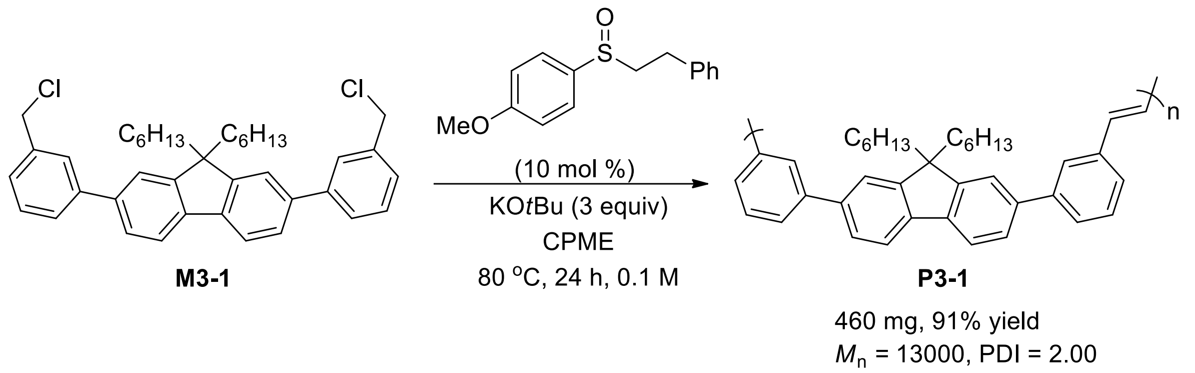

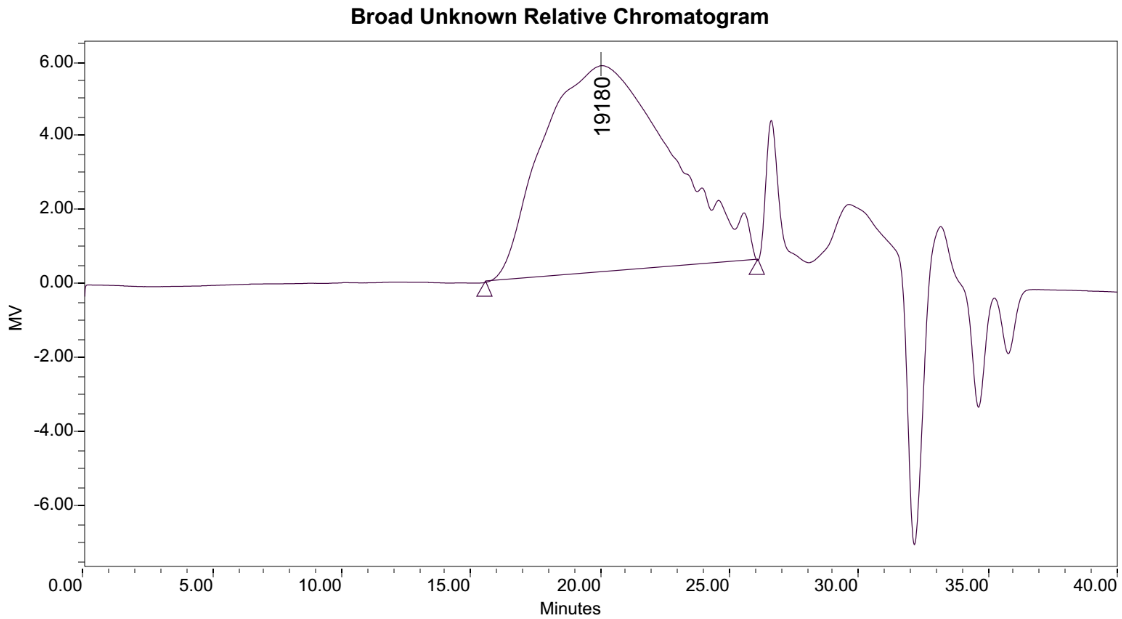

we

Broad Unknown Relative Peak Table

	Distribution Name	Mn (Daltons)	Mw (Daltons)	MP (Daltons)	Mz (Daltons)	Mz+1 (Daltons)	Polydispersity	Mz/Mw	Mz+1/Mw
1		13019	26058	19180	48829	77574	2.001617	1.873866	2.976962

Supplementary Figure 33. Molecular weight of P3-1.

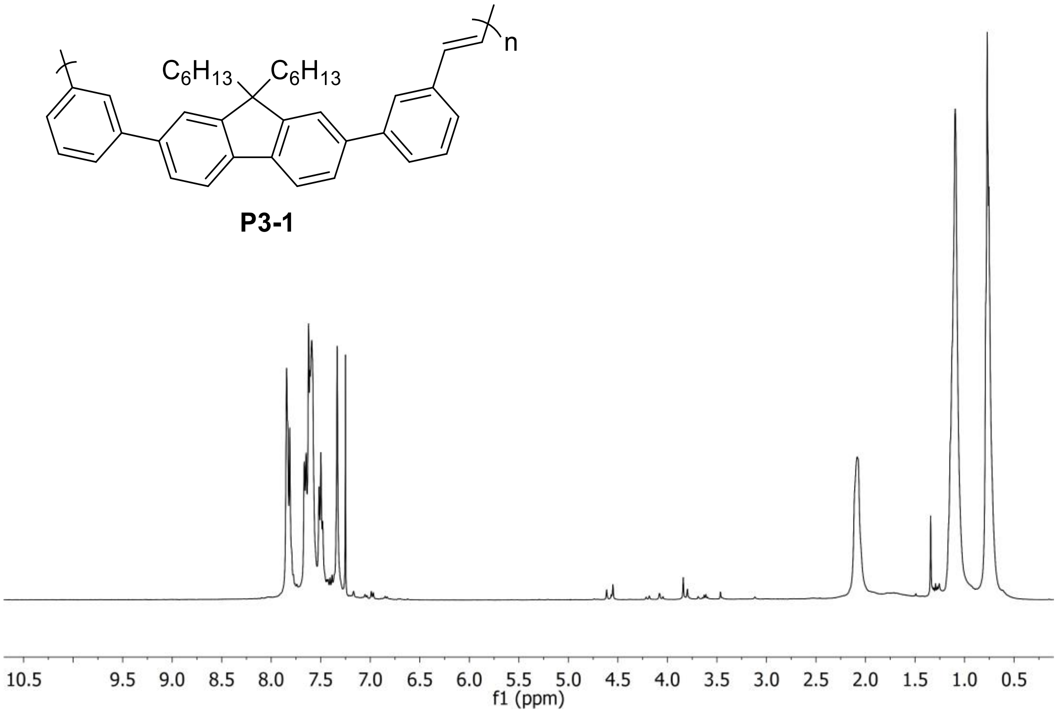

Supplementary Figure 34. ^{1}H NMR spectra of co-polymer P3-1 (^{1}H NMR, $CDCl_3$, 400 MHz).

Synthesis of P4-1.

Co-polymer **P4-1** was synthesized on a 1 mmol scale following the general procedure of the 1 mmol scale BCCP with monomer **M4-1** (545.6 mg, 1 mmol), 1-(phenethylsulfinyl)-4-(trifluoromethyl)benzene (29.8 mg, 0.1 mmol) and KO*t*Bu (336 mg, 3.0 mmol) at 0.1 M concentration. Out of the reaction, co-polymer **P4-1** was obtained as pale yellow solid in 439 mg, 93% yield.

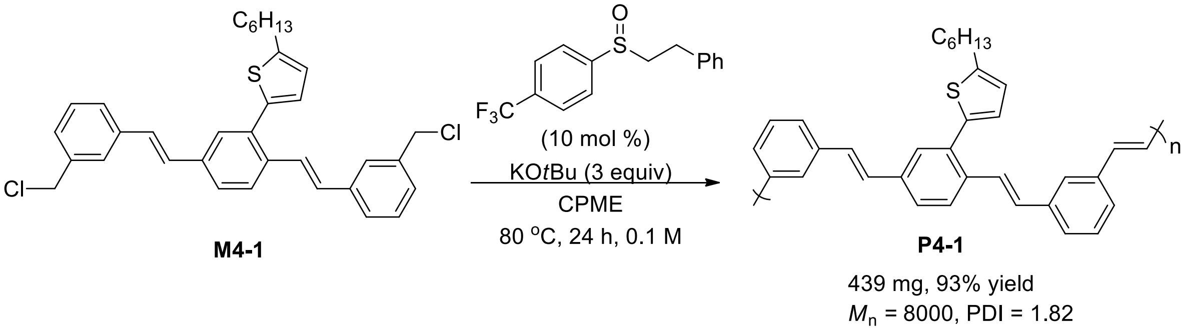

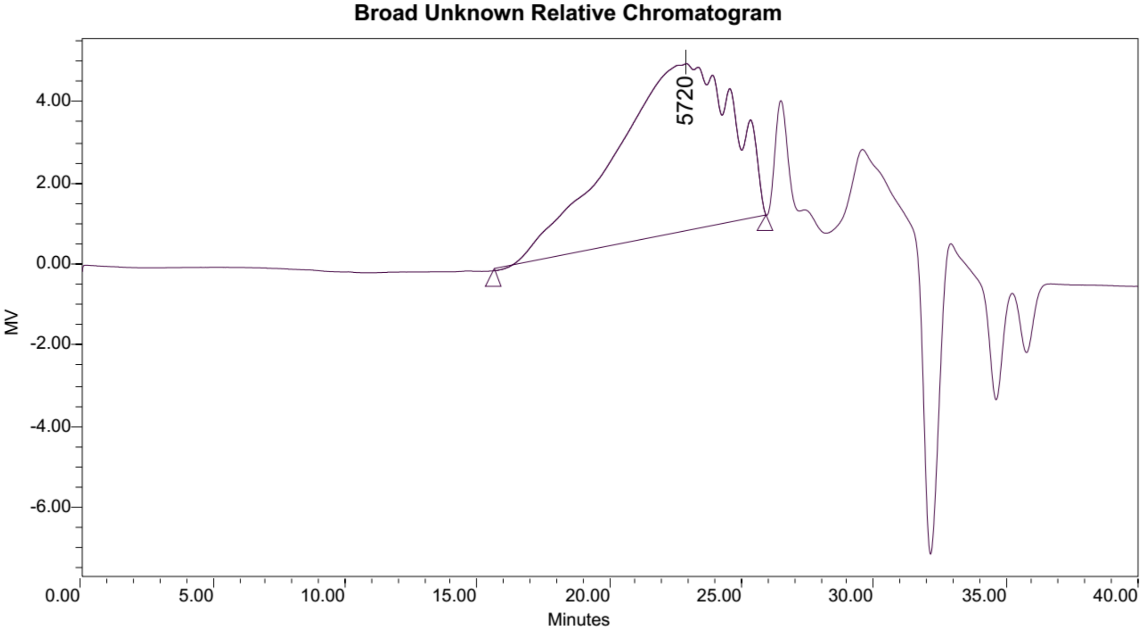

Broad Unknown Relative Peak Table

	Distribution Name	Mn (Daltons)	Mw (Daltons)	MP (Daltons)	Mz (Daltons)	Mz+1 (Daltons)	Polydispersity	Mz/Mw	Mz+1/Mw
1		7984	14545	5720	31573	56836	1.821881	2.170697	3.907588

Supplementary Figure 35. Molecular weight of P4-1.

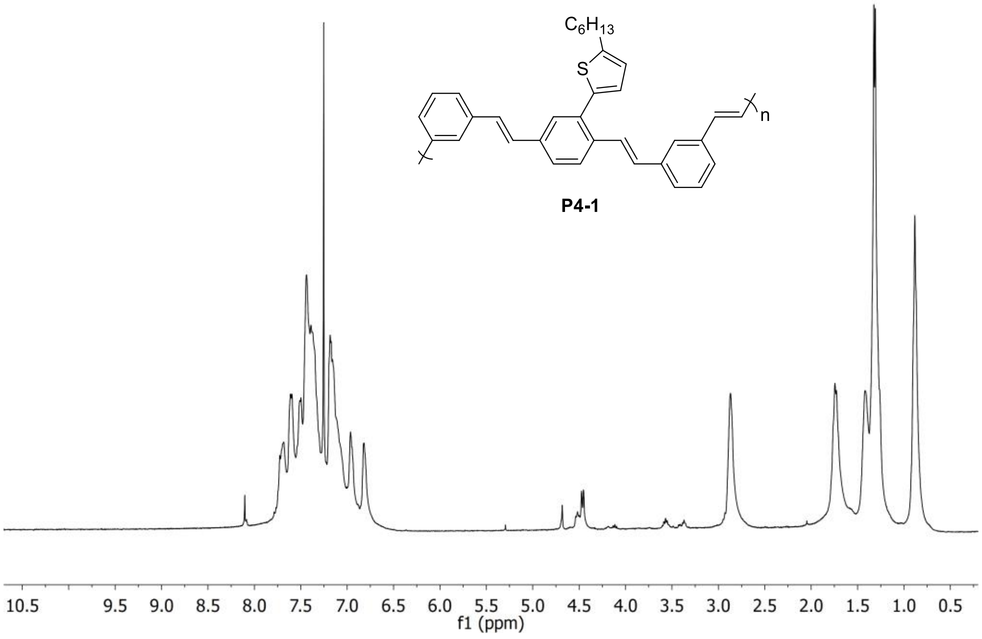

Supplementary Figure 36. ^{1}H NMR spectra of co-polymer P4-1 (^{1}H NMR, $CDCl_3$, 400 MHz).

Characeterization of thermal, photophysical, electrochemical and charge transport properties of P3-1 and P4-1.

General Information

UV-Vis absorption spectra were recorded on a Shimadzu UV-2500 spectrophotometer. Photoluminescence (PL) spectra were measured on a Hitachi F-4600 fluorescence spectrophotometer. Thermogravimetric analysis (TGA) was carried out using a NETZSCH STA 449C instrument. The thermal stability of the samples under a nitrogen atmosphere was determined by measuring their weight loss while heating at a rate of 20 ^{o}C min^{-1} from 25 to 500-600 ^{o}C. Differential scanning calorimetry (DSC) was performed on a NETZSCH DSC 200 PC unit at a heating rate of 10 ^{o}C min^{-1} from 40 to 200-300 ^{o}C under a nitrogen atmosphere. The glass transition temperature (T_g) was determined from the second heating scan. Cyclic voltammetry (CV) was measured in nitrogen-purged dichloromethane for oxidation scan using a CHI voltammetric analyzer. Tetrabutylammonium hexafluorophosphate ($TBAPF_6$) (0.1 M) was used as a supporting electrolyte. The conventional three-electrode configuration is employed, which consists of a platinum working electrode, a platinum wire auxiliary electrode and an Ag wire pseudo-reference electrode with ferrocenium-ferrocene (Fc^+/Fc) as the internal standard. Cyclic voltammograms were obtained at a scan rate of 100 mV s^{-1}. The onset potential of the new compounds was determined from the intersection of two tangents drawn at rising and background current of the cyclic voltammongram at first circle. The half-wave potential ($E_{1/2}$) value for Fc^+/Fc are calculated as the average of cyclic voltammetric anodic and cathodic peaks. The HOMO energy levels were calculated from the oxidation curves according to the formula: - [4.8 eV + (E_{onset} - $E_{1/2(Fc/Fc^+)}$)]. The LUMO energy level was deduced from the energy band gap (E_g) and HOMO level.

Hole mobility were measured using the space charge limited current (SCLC) method. Device structures are ITO/PEDOT:PSS/Polymers/Au for hole-only devices. The SCLC mobilities were calculated by MOTT-Gurney equation. [1,2]

$$J = \frac{9\varepsilon_r \varepsilon_0 \mu V^2}{8L^3}$$

Where J is the current density, ε_r is the relative dielectric constant of active layer material usually 2-4 for organic semiconductors, herein we use a relative dielectric constant of 4, ε_0 is the permittivity of empty space, μ is the mobility of hole or electron and L is the thickness of the active layer, V is the internal voltage in the device, and V = Vapp-Vbi, where Vapp is the voltage applied to the device, and Vbi is the built-in voltage resulting from the relative work function difference between the two electrodes (in the hole-only and the electron-only devices, the Vbi values are 0.2 V and 0 V respectively).

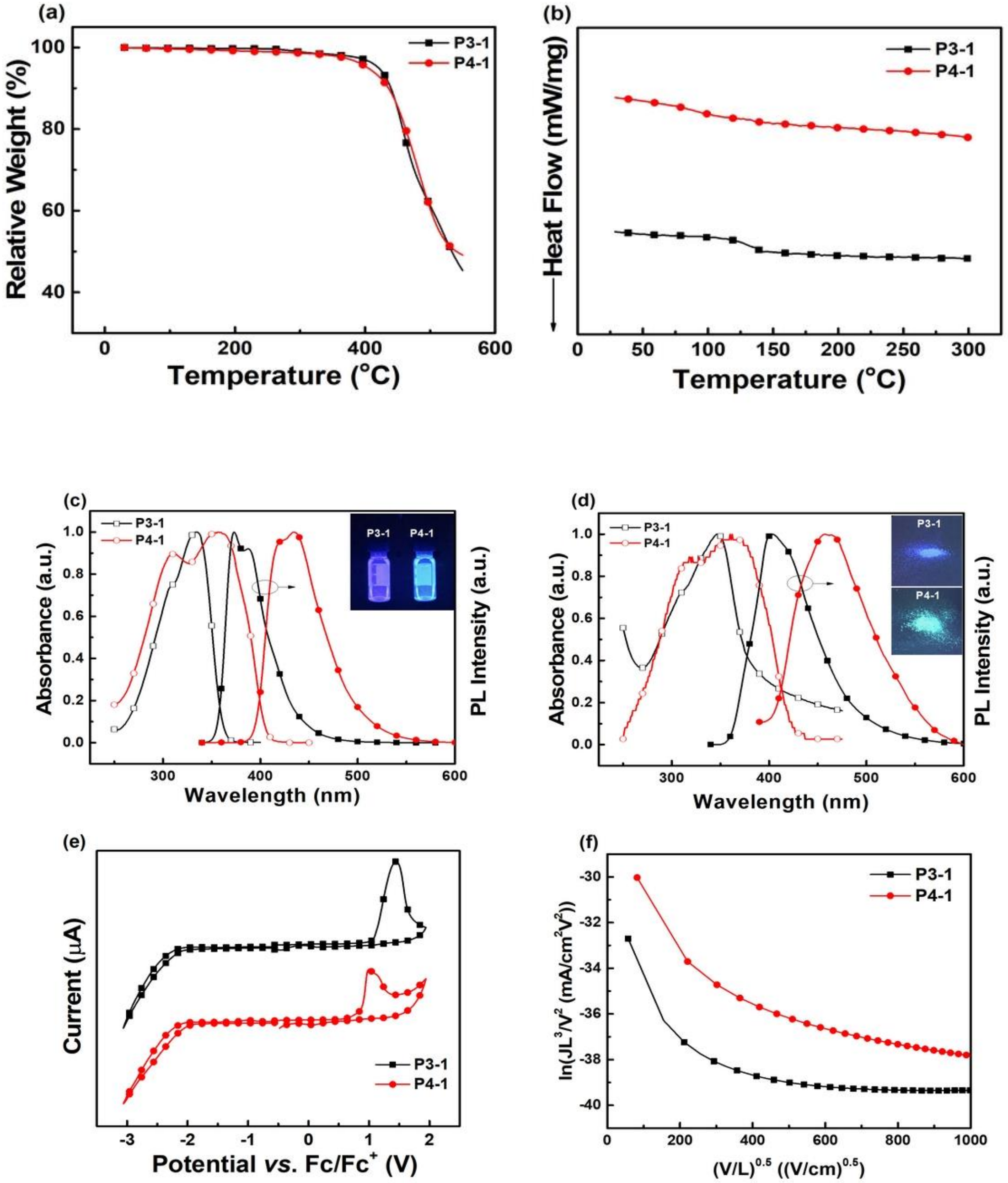

Supplementary Figure 37. (a) TGA curves of the co-polymers P3-1 and P4-1. (b) DSC curves of the co-polymers P3-1 and P4-1. (c) Normalized UV-Vis absorption and PL spectra of co-polymers P3-1 and P4-1 in THF solution and photographs of polymer photoluminescence under 365 nm light in THF solution. (d) Normalized UV-Vis absorption and PL spectra of co-polymers P3-1 and P4-1 in film state and photographs of polymer photoluminescence under 365 nm light in solid state. (e) Cyclic voltammograms of the oxidation curves in dichloromethane and reduction curves in THF for co-polymers P3-1 and P4-1. (f) Current Density-Voltage (J-V) characteristics of hole-only devices with structure of ITO/PEDOT:PSS(30 nm)/P3-1 or P4-1 (30 nm)/MoO3 (8 nm)/Ag.

Supplementary References

(1) Blom, P. W. M. et al. Electric-field and temperature dependence of the hole mobility in poly(p-phenylene vinylene). Phys. Rev. B 55, R656-R659 (1997).

(2) Malliaras, G. G. et al. Electrical characteristics and efficiency of single-layer organic light emitting diodes. Phys. Rev. B 58, R13411-R13414 (1998).
